# Alkyl Tail Variation on Chalcone‐Based Quaternary Pyridinium Salts as Rule‐of‐Thumb for Antimicrobial Activity

**DOI:** 10.1002/ardp.70003

**Published:** 2025-05-11

**Authors:** Francesca Seghetti, Riccardo Ocello, Alessandra Bisi, Matteo Masetti, Silvia Gobbi, Federico Falchi, Giovanna Angela Gentilomi, Francesca Bonvicini, Federica Belluti

**Affiliations:** ^1^ Department of Pharmacy and Biotechnology Alma Mater Studiorum‐University of Bologna Bologna Italy; ^2^ Computational and Chemical Biology Italian Institute of Technology IIT Genoa Italy; ^3^ Microbiology Unit IRCCS Azienda Ospedaliero‐Universitaria di Bologna Bologna Italy

**Keywords:** antibacterial and antifungal effects, cell membrane interaction, chalcone scaffold, molecular dynamics simulation, quaternary alkyl‐pyridinium salts

## Abstract

Aiming at developing a new class of quaternary pyridinium salts, the lead compound **1**, characterized by a pyridine‐3‐yl chalcone framework, was rationally modified by inserting alkyl functions varying from 6 to 18 carbon units. Among the set, some valuable lead compounds were identified. Derivatives **4**–**6** were primarily active against *Staphylococcus aureus* and *Candida albicans*, respectively (MIC = 1.56 and 3.125 μM). In comparison, analogs **4** and **5** showed significant activities against *Escherichia coli* (MIC = 6.25 μM). Interestingly, the antimicrobial property of compounds **4**–**6**, as well as their antibiofilm activity, occurred at lower concentrations than their cyto‐ and erythrocyte toxicities, thus ensuring a favorable safety profile. Structure–activity relationship analysis highlighted the critical role of the alkyl tail length in the antimicrobial activity, and optimal results were observed for moieties ranging from 10 to 14 carbon units. Molecular dynamics studies performed on **2** and **5** by modeling them on Gram‐positive and Gram‐negative membranes showed that the derivatives, upon diffusing across periodic boundary conditions, were able to intercalate into the microbial membranes. The difference in diffusion rates provides useful information to support the diverse antimicrobial potencies of the newly designed quaternary pyridinium salt.

## Graphical abstract

A small library of quaternary pyridinium salts was developed as membrane‐interacting antimicrobials. In particular, analogs **4–6** effectively inhibited *Staphylococcus aureus* growth, while **4** and **5** were active against *Escherichia coli*. Molecular dynamics studies supported the capability of derivative **5** to efficiently interact with the Gram‐positive membrane, corroborating the experimental antimicrobial data.

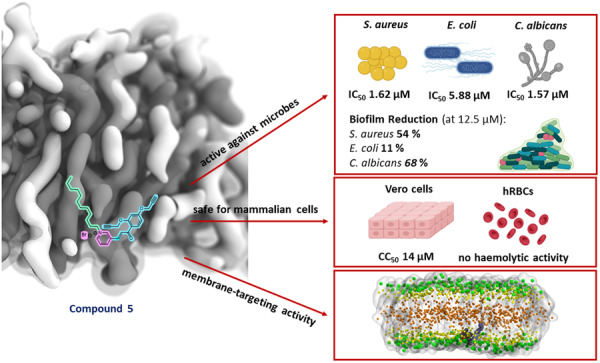

## 1 Introduction

Due to the significant virulence and high incidence, clinical management of microbial infections represents a serious and growing problem in global healthcare; some daunting issues, among which the escalating occurrence of microbial resistance to traditional antibiotics and the formation of biofilms, contributed to the development of chronic infections [1, 2], thus underscoring the urgent need to develop new, safe, and effective treatment strategies [3].

In the past two decades, the microbial membrane has emerged as a crucial component for cell survival; its targeting by membrane‐active compounds offers promise for avoiding toxicity and induction of resistance phenomena, thus attracting consistent attention and leading to the development of several series of membrane‐active antimicrobial agents [4].

Biological membranes of bacteria and fungi are regarded as validated targets for the development of antimicrobial therapeutics because the surface topology and biochemical architecture of the membranes, as well as the chemical composition, hydrophobicity, and charge, differ significantly between microbial targets and mammalian cells. Specifically, the bacterial membrane comprises 75% neutral phospholipids and 25% anionic phospholipids, while the eukaryotic membrane consists of only neutral phospholipids [5, 6, 7]; this feature plays a remarkable role in the interaction between molecules and phospholipid bilayers, thus enabling the design of antimicrobials without undesirable toxic effects on host cells. Cell membranes predominantly composed of phosphatidylglycerol (PG), phosphatidylethanolamine (PE), and cardiolipin (CL), are found in many bacterial pathogens; in particular, in the cytoplasmic membrane of Gram‐positive bacteria and both the outer membrane (OM) and the inner membrane (IM) of Gram‐negative bacteria. The slow porin‐mediated uptake across the OM and active efflux via efflux pumps in the IM creates a permeability barrier to small molecules, including the antibacterial agents [8]. As for fungi, the presence of a sterol‐based biomolecule, ergosterol (ERG), in the lipid bilayer strongly characterizes the structural properties of these membranes [9]. A valuable family of antimicrobial agents is represented by the class of quaternary ammonium compounds (QACs). They have been used for over a century to prevent microbial infections in hospitals and in various consumer products, along with treating human infections due to their wide spectrum of activity against bacteria and fungi [10]. It is commonly thought that they act by targeting biological membranes as a universally acknowledged mechanism of action [11]. Indeed, QACs, amphiphile molecules comprising a hydrophilic quaternary nitrogen atom and a hydrophobic alkyl function (representing the cationic head group and the non‐polar tail, respectively) that can exert biocidal effects against bacteria and fungi by mimicking membrane properties. One of the different hypotheses elaborated on the mechanism of action of QACs suggests damage to the cell membrane surface, caused by a combined hydrophobic and electrostatic adsorption phenomenon, ultimately leading to its lysis. In particular, the positively charged ammonium function is drawn toward the overall negative charge of the bacterial cell membrane; this anchoring step is essential as it sets the basis for the subsequent cell disruption, which is the consequence of the insertion of the aliphatic QAC portion into the bacterial cell membrane [11]. This peculiar mechanism accounts for the wide activity spectrum of this class of compounds, and it also allows to selectively affect the integrity of prokaryotic cells over their eukaryotic counterpart. This selectivity relies on the composition of mammal membranes, principally composed of zwitterionic lipids that account for a low interaction with QACs, different from pathogens’ membranes, rich in negatively charged components and highly affine for positively charged molecules. Recently, a newly proposed mechanism of QACs' action reported their capability of reducing the stored membrane curvature elastic stress, which leads to functionality loss of different membrane‐associated proteins [12, 13]. A large variety of QACs have been developed aimed at performing structure–activity‐relationship (SAR) studies [14], from which it has emerged that alkyl chain length regulates the antimicrobial effect and, generally, the aliphatic tail composed of 12–16 carbon units yielded superior results. In particular, the maximum effect against Gram‐negative pathogens was observed for analogs bearing a 12–14 carbon (C12–C14) unit, whereas Gram‐positive growth was inhibited by analogs with a longer tail (C14–C16) [13]. QACs characterized by decreased electron density in the cationic head are supposed to interact tightly with the polarizable cell wall elements, namely membrane phospholipids, and elicit a remarkable antimicrobial effect. Moreover, well‐balanced hydrophilic–lipophilic properties were a key requirement for best‐performing QACs.

**Figure 1:**
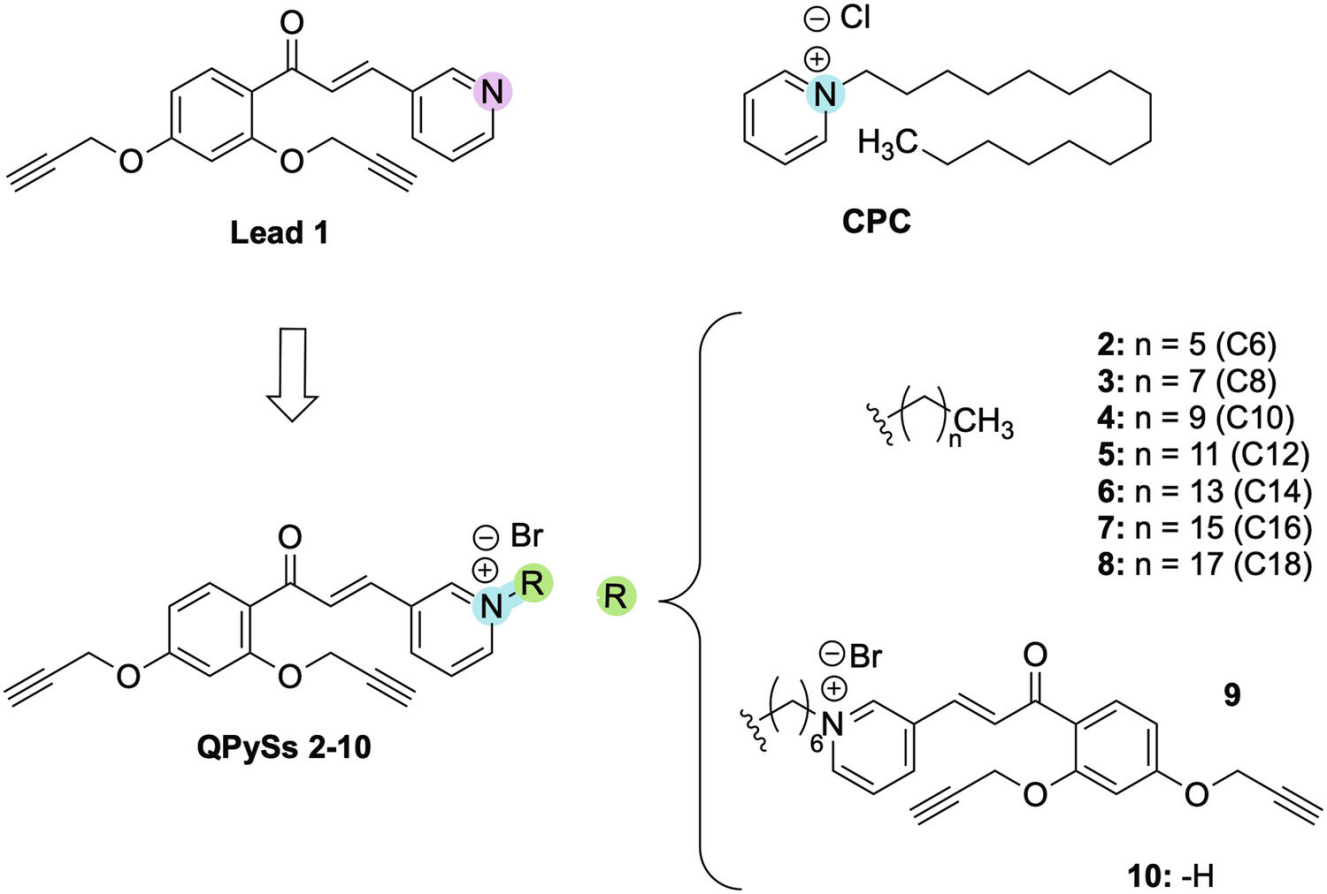
Design strategy. Functionalisation of the chalcone‐based analog **1** to obtain QPySs **2–10** and the structure of cetylpyridinium chloride (CPC).

Cetylpyridinium chloride (CPC, Figure 1), a Quaternary Pyridinium Salt (QPyS) widely used as an antiseptic in dental care products, was initially reported in 1939 for its antibacterial activity [15]. In the literature, there are numerous examples of QPySs as antimicrobial agents [4]. Particularly, this class of compounds, endowed with a flat cationic pyridine moiety, showed an enhanced affinity for biological membranes, being able to favorably interact with the membrane's anionic components, thus resulting in effective disruption of the microorganisms’ defence system [16]. The molecular basis underpinning the QPySs’ antibacterial activity has been deeply investigated, and different modes of action have been proposed. In general, the interaction of QPyS positive charge with cytoplasmic membrane phospholipids results in distortion and disruption of the membrane and interference with cell damage and leakage [16, 17].

Naturally occurring chalcones (1,3‐diaryl‐2‐propen‐1‐ones), a class of secondary metabolites and precursors of flavonoids, are multifunctional compounds as they show a pleiotropic effect, including antioxidant, anti‐inflammatory, and antimicrobial, as extensively reported in many reviews [18, 19]. On this basis, the chalcone scaffold is recognized as a privileged structure, able to interact with a wide range of molecular targets, and an appropriate chemical platform suitable to be purposely modified to obtain, upon properly addressed modification, libraries of compounds with distinct bioactivity profiles [20, 21, 22, 23].

This study deals with the design, synthesis, biological evaluations, and molecular dynamics (MD) studies of a new class of aliphatic QPySs based on chalcone scaffold as membrane‐targeting antimicrobial agents.

The aim of the study was the development of QPySs inspired by our lead **1** (Figure 1), previously reported to have antifungal and antileishmanial effects (IC_50_ values of 43.8 μM against *Candida albican*s and 4.0 μM against *Leishmania donovani*) [24, 25]. This compound also showed a slight capability to affect *Staphylococcus aureus* growth (IC_50_ = 47.65 μM), but it was unable to inhibit *Escherichia coli* (MIC > 100 μM). A remarkable cytotoxic effect (CC_50_ = 13.0 µM) represented the primary drawback of analog **1**. Aimed at widening the activity spectrum and improving the selectivity index (SI) of this lead, thus ensuring a safety profile, a properly addressed modification was performed to focus on targeting the microbial membrane, a crucial element for bacterial and fungal survival.

In detail, a series of new compounds was designed in which the pyridine ring of **1** was converted into a QPyS through its functionalization with a linear alkyl moiety. The advantage of this class of quaternary compounds lies in the absence of toxic side effects on mammalian cells through a selective mechanism of action against microbial membranes. In line with the reported role of lipophilic moieties in conferring antimicrobial properties, the pyridine cationic charge could allow electrostatic interactions with the negatively charged bacterial cell membranes, while the aliphatic tail could drive the insertion of the molecule into the lipid bilayer with consequent cell damage.

A small library of QPySs (compounds **2–9**, Figure 1) was designed in which the hydrophobicity of the aliphatic portion was progressively increased, varying its length from 6 to 18 carbon units to perform a SAR study. Moreover, to evaluate the contribution of the lipophilic tail, two prototypes were also designed: a positively charged pyridinium analog (**9**) and a bis‐cationic chalcone (**10**) with two net positive charges. Finally, to assess the role of the chalcone scaffold, a C14‐functionalised 3‐methylpyridine (**11**) was synthesized.

The obtained QPyS analogs were evaluated for their antimicrobial potential toward Gram‐positive, Gram‐negative, and fungi, as well as their cytotoxicity against epithelial cells. The best‐performing analogs were deeply studied, and the effect on biofilm formation, along with the hemolytic properties, was investigated. In addition, MD simulations allowed shedding light on the affinity of two selected compounds for the pathogen membranes.

## 2 Results and Discussion

### 2.1 Chemistry

**Scheme 1:**
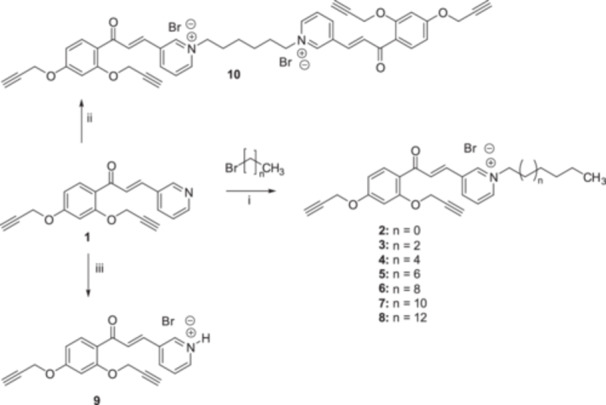
Synthesis of chalcone‐based QPySs **2–10**. Reagents and conditions: (i) selected 1‐Br‐alkane, ACN, 80°C reflux; (ii) 1,6‐dibromohexane, ACN, 80°C; (iii) HBr, EtOH, rt.

**Scheme 2:**
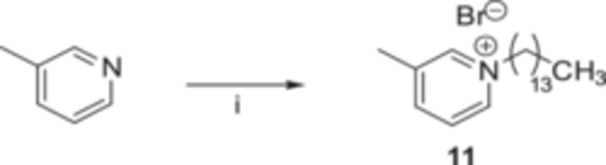
Synthesis of 3‐methylpyridine‐based analog **11**. Reagents and conditions: (i) 1‐bromotetradecane, ACN, 80°C.

The designed QPySs **2–9** and **11** were synthesized by exploiting the Menshutkin‐type reaction between compound **1** [24] and different alkyl bromides (**2–9**, Scheme 1) or between 3‐methylpyridine and 1‐Br‐tetradecane (**11**, Scheme 2) [26]. In contrast, analog **9** was obtained by HBr treatment of **1** (Scheme 1).

### 2.2 Pharmacology/Biology

The antimicrobial activity of the newly designed compounds (**2**–**11**) was evaluated against three reference strains, including *S. aureus* ATCC 25923, *E. coli* ATCC 25922, and *C. albicans* ATCC 10231. Since the lack of selectivity and the cytotoxicity represent a limiting parameter in the drug discovery pipeline, the overall safety profile was determined In Vitro by measuring the viability of Vero cells and the detrimental effect on the structure of human red blood cells (hRBCs) membrane. Specifically, the hemolytic assay is a recognized method for the evaluation of the lytic interactions with the mammalian membranes, thus providing information on the therapeutic value of the compounds.

#### 2.2.1 In Vitro Antimicrobial Activity Study

The antimicrobial activity of compounds **2–11** was assayed In Vitro by a standardized microdilution method, and the results, expressed as MIC (minimum inhibitory concentration) and IC_50_ (concentration giving rise to a 50% inhibition), are listed in Table 1.

**Table 1:** MIC and IC_50_ (expressed as µM) of the QPySs 2–11 and the lead compound.

		MIC, IC_50_ [95% confidence interval]		
Comp.	Carbon units	*Staphylococcus aureus*	*Escherichia coli*	*Candida albicans*
**1**	/	100	> 100	100^a^
		47.65 [38.14–69.06]	n.d.	43.8 [30.6–62.7]
**2**	6	50	> 100	100
		34.35 [32.96–35.81]	n.d.	68.73 [61.47–76.85]
**3**	8	6.25	12.5	50
		2.65 [2.41–2.91]	10.95 [10.65–11.25]	30.49 [28.16–33.01]
**4**	10	1.56	6.25	6.25
		0.84 [0.24–2.97]	4.48 [3.57–5.39]	4.28 [2.80–6.49]
**5**	12	3.125	6.25	3.125
		1.62 [1.55–1.69]	5.88 [5.69–6.08]	1.57 [1.47–1.67]
**6**	14	3.125	50	1.56
		1.30 [1.18–1.44]	36.33 [29.95–44.07]	1.32 [1.01–1.72]
**7**	16	12.5	> 100	6.25
		4.21 [3.38–5.23]	n.d.	5.76 [4.21–5.98]
**8**	18	100	> 100	> 100
		14.23 [12.56–16.12]	n.d.	52.45 [11.36–100]
**9**	/	> 100	> 100	> 100
		n.d.	n.d.	n.d.
**10**	6	50	50	100
		58.34 [25.76–100]	46.00 [44.83–46.23]	95.00 [90.92–97.82]
**11**	14	6.25	12.5	6.25
		5.89 [5.80–6.04]	7.39 [3.71–14.71]	5.43 [4.39–6.71]
GEN	/	6.54	13.09	n.d.
		4.0 [3.97–4.05]	6.11 [5.03–7.27]	n.d.
FLC	/	n.d.	n.d.	0.82
		n.d.	n.d.	0.23 [0.21–0.25]

Abbreviations: FLC, fluconazole; GEN, gentamicin; n.d., not determined.

^a^ Previously reported in reference [24].

Compounds **2–8** exhibited a broad spectrum of activity against the tested strains, being able to inhibit microbial growth to a different extent. Taking into account MIC values, this set showed excellent inhibitory properties against *S. aureus* and *C. albicans* with active compounds up to 1.56 µM (**4** and **6**, respectively) and moderate activity against *E. coli* with two active compounds at 6.25 µM (**4** and **5**). In detail, considering the IC_50_ values, compounds **2–8** turned out to affect *S. aureus* growth in the 0.84–34.35 μM range, with **4** being the most potent and **2** the least active; on the contrary, regarding the inhibitory effects on *C. albicans*, the 1.32–68.73 μM activity range was recorded (**6** as the most potent and **2** the least active). Due to the membrane composition, Gram‐negative strains are more challenging to affect than Gram‐positive ones, and only the sub‐set **3–6** proved effective against *E. coli* in the 4.48–36.33 μM range; again, **4** was the most potent and **6** the least active. The overall higher antimicrobial potency of the QPySs **2**–**8** on *S. aureus* and *C. albicans* compared with *E. coli* can be ascribed to the OM barrier.

**Figure 2:**
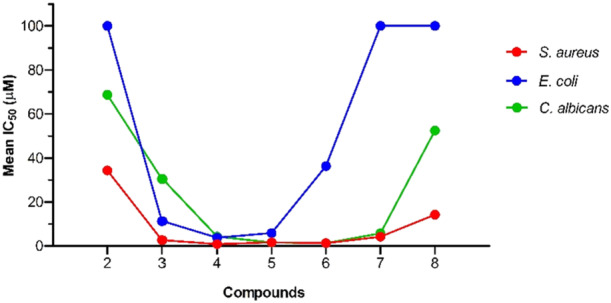
Mean IC_50_ values of the analogs **2**–**8** obtained for the three tested strains.

In general, regardless of the tested strains, it is possible to envisage a common behavior in the antimicrobial effectiveness of the compounds **2**–**8** that is related to the length of the alkyl chain in their structure. As shown in Figure 2, IC_50_ values decreased with the length of the aliphatic portion to a maximum potency of 1.3 μM for *S. aureus* and *C. albicans* and 3.7 μM for *E. coli*; thereafter, the values increased, demonstrating an overall lower antimicrobial activity of the compounds with longer alkyl tails. Based on the present data, the dependence is non‐linear, showing extremes in the activity for analogs with the C10–C14 side chain. The correlation between antimicrobial efficiency and chain length observed for *S. aureus* is consistent with data obtained from other studies [27, 28]. A low antimicrobial activity was noticed for the chalcones with a C6 and C8 tail, likely due to a weak hydrophobic interaction with the lipid membranes. An analogous behavior was detected for the longer C16 and C18 analogs, whose strong lipophilic character could reduce their solubility, thereby hampering their transport through the cell membranes [29].

The effect of the number of carbons in the QPySs tail is well displayed by the plots of the dose–response curves produced for the compounds on the reference strains (Supporting Information S2: Figure S1), where it clearly emerges that the antimicrobial potency is strictly dependent on the chain length, regardless of the tested model system. As a proof of concept, compound **9** without an alkyl chain and the bis‐cationic **10** were assayed toward the three selected strains, and they displayed weak antimicrobial activities (MIC ≥ 50 µM), thus indicating that a simple alkyl tail is a mandatory structural parameter that rules the antimicrobial activity. In addition, the C14‐functionalized 3‐methylpyridine **11** demonstrated a moderate inhibitory effect with MIC values in the range 6.25–12.5 µM, thus corroborating the contribution of the chalcone framework in the overall antimicrobial activity.

#### 2.2.2 Colistin Association Assay

**Figure 3:**
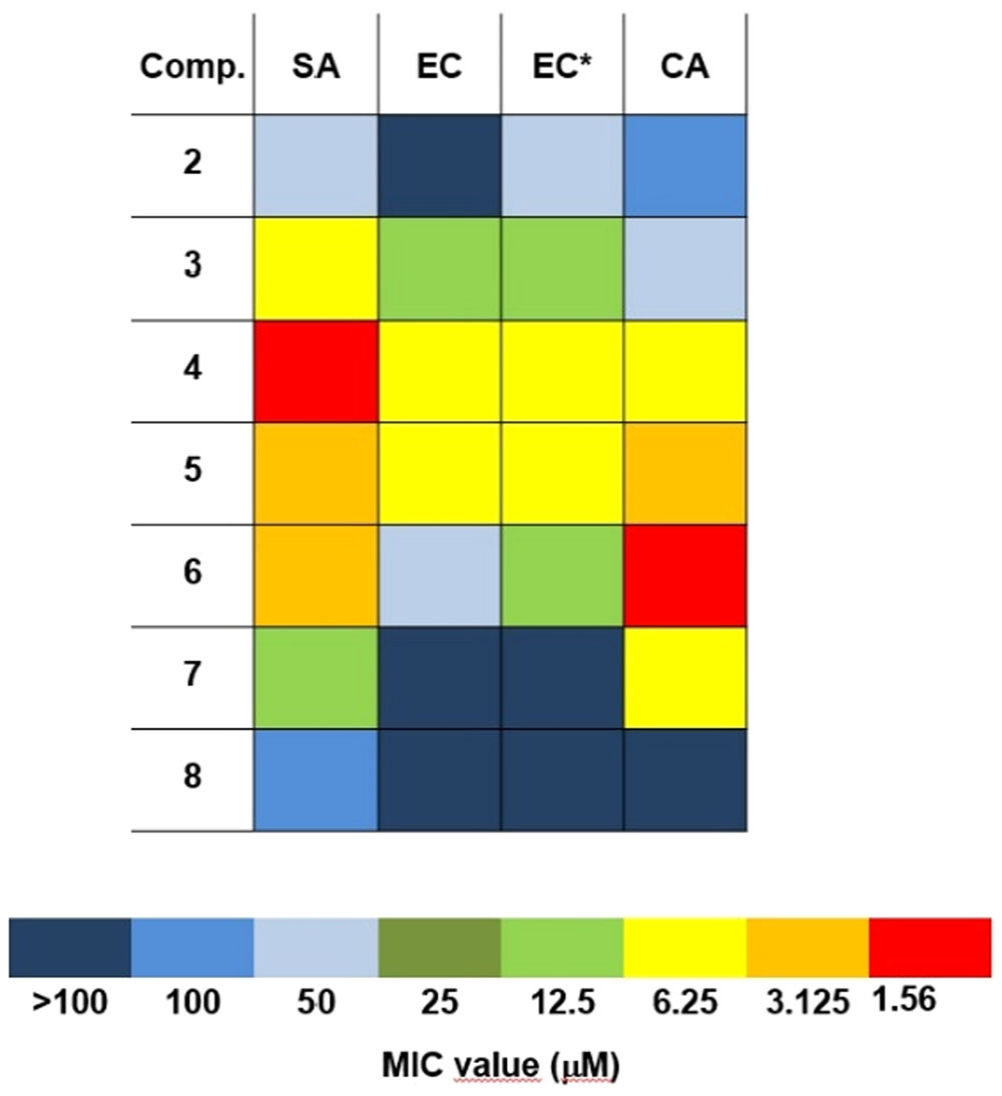
Heatmap with MIC values of the analogs **2**–**8** for *Staphylococcus aureus* (SA), *Escherichia coli* (EC), and *Candida albicans* (CA). As for *E. coli*, data are presented as obtained by testing the QPySs alone, and in association with colistin (EC*).

To shed light on the different activities of compounds **2**–**8** against the two bacterial strains, they were assayed in the range of concentration 100–0.19 µM in association with colistin, a conventional OM‐damaging antibiotic, at a sub‐inhibitory concentration (MIC/8 = 0.027 µM). The OM perturbation caused by colistin sensitized *E. coli* to analogs **2** and **6**; in particular, the potency of **2** was completely restored to the same extent measured for *S. aureus* (MIC = 50 µM), and the effectiveness of compound **6** showed a fourfold MIC decrease (Figure 3). This is a confirmation of the ability of these new QPySs to also affect Gram‐negative proliferation once they pass through the OM.

#### 2.2.3 In Vitro Cytotoxicity Study

The evaluation of the viability of Vero cells, a non‐malignant epithelial cell line, was included in the screening pipeline of the synthesized library, as the intrinsic cytotoxicity of the compounds is a crucial challenge to the progress of antimicrobial agents in clinical development. In this study, the effect of compounds **2–11** on cell metabolism was measured after a treatment of 48 h, and data were expressed as CC_50_ values (Table 2). Results demonstrated that analogs **2–8** affected Vero cells' viability, and that the cytotoxicity gradually increased in relation to the length of the alkyl chain, unlike the observed antimicrobial trend. The cationic analog **9**, lacking the alkyl chain, displayed a high cytotoxicity (CC_50_ = 6.20 µM), while the C14‐functionalized 3‐methylpyridine **11** showed a moderate effect on cell viability (CC_50_ = 24.48 µM).

**Table 2:** CC_50_ (expressed as µM) of the QPySs **2–11** and the lead compound **1**, and SI was measured for the different microbial strains.

	SI^a^			CC_50_ [95% CI]
Compound	*Staphylococcus aureus*	*Escherichia coli*	*Candida albicans*	Vero cells
**1**	< 1	< 1	< 1	13^a^ [11.4–14.8]
**2**	> 2	> 1	> 1	> 100
**3**	17.7	4.3	1.5	47.00 [42.38–47.33]
**4**	15.3	2.9	3.0	12.82 [10.07–16.33]
**5**	8.6	2.4	8.9	14.00 [12.26–16.00]
**6**	4.7	< 1	4.7	6.16 [5.50–6.90]
**7**	1.3	< 1	< 1	5.38 [4.75–6.09]
**8**	< 1	< 1	< 1	5.73 [4.86–6.77]
**9**	< 1	< 1	< 1	6.20 [5.22–7.35]
**10**	> 2	> 2	> 1	> 100
**11**	3.9	3.3	4.5	24.48 [21.65–27.67]
DOX	n.d.	n.d.	n.d.	70.62 [64.84–77.03]

Abbreviation: DOX, doxorubicin.

^a^ Selectivity index (SI = CC_50_/IC_50_).

The SI, a crucial parameter, was calculated by comparing CC_50_ and IC_50_ values (Table 2); the best‐performing analogs **4** and **5**, endowed with a broad spectrum antimicrobial effect, proved to inhibit Vero cell growth with CC_50_ values of 12.82 and 14.00 μM, respectively, showing a selectivity between microbial and human cells, with SI values ranging from 2.4 to 15.3 for the different species. Compound **6** exhibited satisfying selective activity against *S. aureus* and *C. albicans* (SI = 4.7 for both pathogens), but not for *E. coli* (SI < 0.1). Comparing the SI values of compounds **6** and **11**, both bearing a C14 tail, it is possible to assume the contribution of the chalcone motif in the antimicrobial activity against *S. aureus* and *C. albicans*.

#### 2.2.4 In Vitro Hemolytic Activity

**Figure 4:**
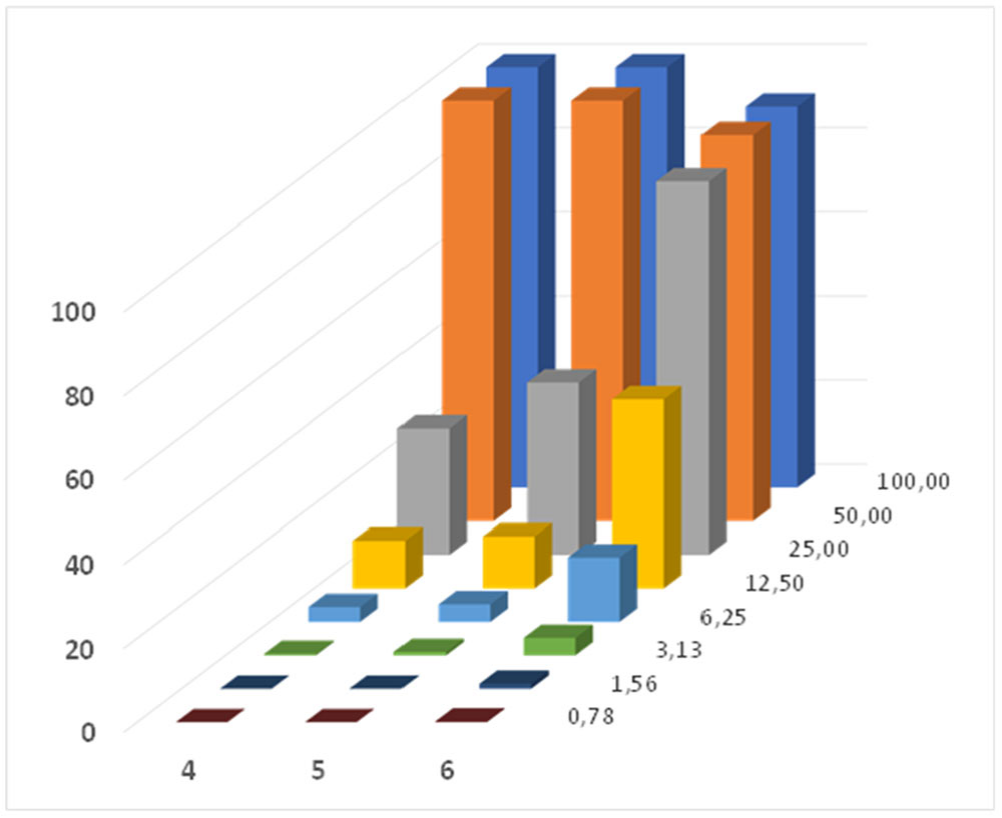
Mean percentage values of the hemolytic activity of analogs **4**–**6** at different concentrations (0.78–100 µM).

The best‐performing compounds **4–6** were further investigated for their hemolytic effect on hRBCs. Erythrocytes, which lack internal organelles, are the most widely used cell membrane systems to evaluate compound–membrane interactions [30]. Moreover, they provide information concerning the therapeutic value and systemic toxicity of drugs. Results are presented in Figure 4.

The QPySs showed a dose‐dependent hemolytic activity that was systematically affected by hydrophobicity as it increased with the alkyl chain elongation, even in this limited range of analyzed compounds (**4–6**: C10‐C14 alkyl tail carbon units). This trend was particularly evident at the concentration of 25 µM, where the hemolytic activity was 30.0% for **4**, 40.9% for **5**, and 88.7% for **6**. Notably, the concentrations at which hemolysis was observed always far exceeded the MIC values of the compounds, thus, once again, confirming their safety on mammalian cells.

#### 2.2.5 Antibiofilm Activity

Having demonstrated the high antimicrobial potency of **4–6**, together with their safety profile on mammalian cells, they were further investigated to determine their antibiofilm capacity in terms of inhibition of biofilm production (Supporting Information S2: Figure S2). Among the three tested compounds, **4** exhibited the highest activity against the production of *S. aureus* and *E. coli* biofilms, with percentage values > 90% at 25 µM. As for *C. albicans*, a strong antibiofilm activity was demonstrated for **6** up to 3.125 µM with a reduction in the biofilm formation of 68.3%.

### 2.3 MD Simulations

To investigate the mechanism by which the newly designed QPySs interact with the bacterial cell membranes and to discern potential differences in the behavior between Gram‐positive and Gram‐negative pathogens, MD simulations were conducted on molecules **2** and **5** with a C6 and C12 tail, respectively, characterized by different antimicrobial potencies.

**Figure 5:**
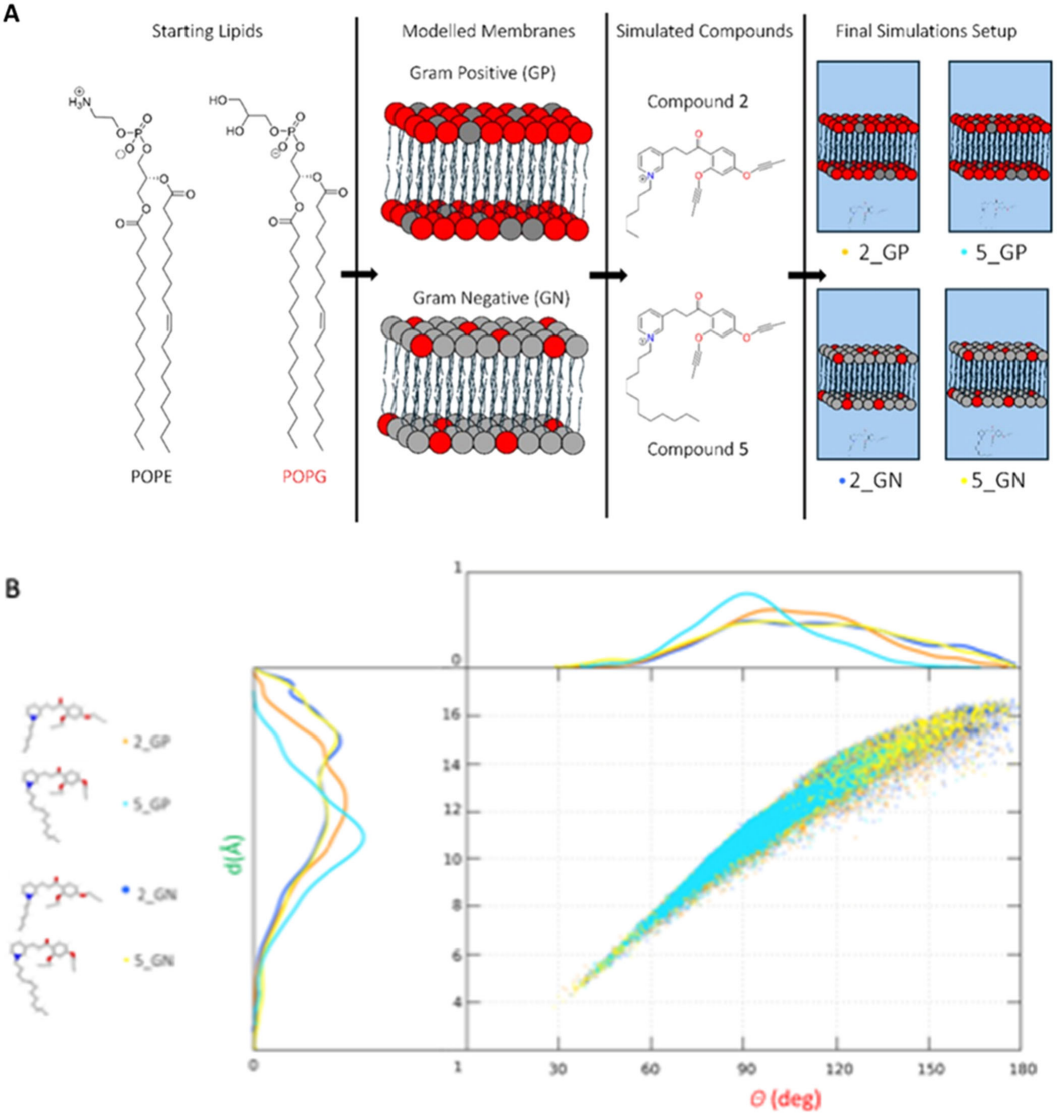
(A) Schematic representation of the simulation procedure. Structural 3D representation of the four simulated systems, differentiated by the lipidic composition ratio [Gram‐positive (GP) and Gram‐negative (GN) membranes] and molecules **2** and **5**. (B) Scatter plot distribution of head‐to‐tail distance and the head‐to‐tail angle sampled during the simulation course. Analogs **2** and **5** in the GP membrane are the orange and cyan dots, respectively; molecules **2** and **5** in the GN membrane are the blue and yellow dots, respectively. On the left and top edges of the 2D graph, the linear distributions of distances and angles are projected using single line traces consistently with previously reported colors.

Two membrane models were employed: one representing the membrane of *S. aureus* and another representing the IM of *E. coli*. The choice to focus on *E. coli* IM was motivated by its potential to provide deeper insights into the mechanism of action of these QPySs, while also considering computational efficiency. Indeed, the interaction with the IM plays a crucial role in eliciting antimicrobial activity and enables a more consistent comparison with the cell membrane of *S. aureus*. The membrane compositions were modeled based on specific ratios of phospholipids (Figure 5, Panel A), with a 1:3 ratio of 1‐palmitoyl‐2‐oleoyl‐phosphatidylethanolamine (POPE) to palmitoyloleoyl‐phosphatidylglycerol (POPG) for Gram‐positive (GP) *S. aureus‐like* [9], and an inverted ratio of 3:1 of POPE:POPG for Gram‐negative (GN) *E. coli‐like* membranes [31].

MD simulations were performed for each QPyS‐membrane system (**2**_GP, **2**_GN, **5**_GP, **5**_GN) to evaluate the molecular interactions with the membranes and the extent of their intercalation within the membrane structures. Molecular conformations were initially analyzed in terms of geometric parameters (Figure 5, Panel B), such as the head‐to‐tail distance (*d*) and angle (*θ*).

Additionally, density profiles of the double‐layer leaflets were examined to assess QPyS penetration into the membranes, providing further insights into the equilibrium distribution of the studied analogs within the lipid bilayers (Figure 5, Panel B). Considering these variables as relevant and informative enough to obtain a good representation of the molecular conformations, differences in molecular behavior became apparent when plotted together. Among the four developed systems, **5**_GP exhibited a more pronounced tendency to adopt a smaller head‐to‐tail (with a maximum distribution at about 90°) and a smaller average head‐to‐tail distance than **2**_GP. Such differences were less marked in the GN systems. Regardless of the membrane system, both ligands primarily adopted a bent conformation rather than an elongated one, with a significantly smaller angle for compound **5** in the Gram‐positive membrane model. The planar and cationic head remained firmly bound to the polar region of the membrane, engaging transient H‐bonds with the lipid polar headgroups. The difference in charge between the cationic compound and the negatively charged POPG lipids explains the partial insertion of the molecules inside the membrane models.

A deeper investigation of the selected QPySs conformations was then conducted, taking into account all the molecule's dihedral angles and performing the cluster analysis [32]. This approach allowed for rapidly and accurately evaluating the conformational sampling of the studied compounds during MD simulations and comparing their differences when absorbed in Gram‐positive and Gram‐negative membranes. Comparing the results, both QPySs, when interacting with the Gram‐positive membrane, mainly adopted an l‐shaped conformation where the chalcone framework was consistently oriented orthogonally to the alkyl tail. Interestingly, all three main clusters of both molecules intercalated in Gram‐positive membranes showed this behavior, indicating that the analogs were mainly embedded in a similar conformation (Supporting Information S2: Figure S3). In contrast, when the molecules interacted with the Gram‐negative membrane, they showed significant conformational variability, with **2** and **5** showing at least two clusters with either an extended or a bent conformation.

**Figure 6:**
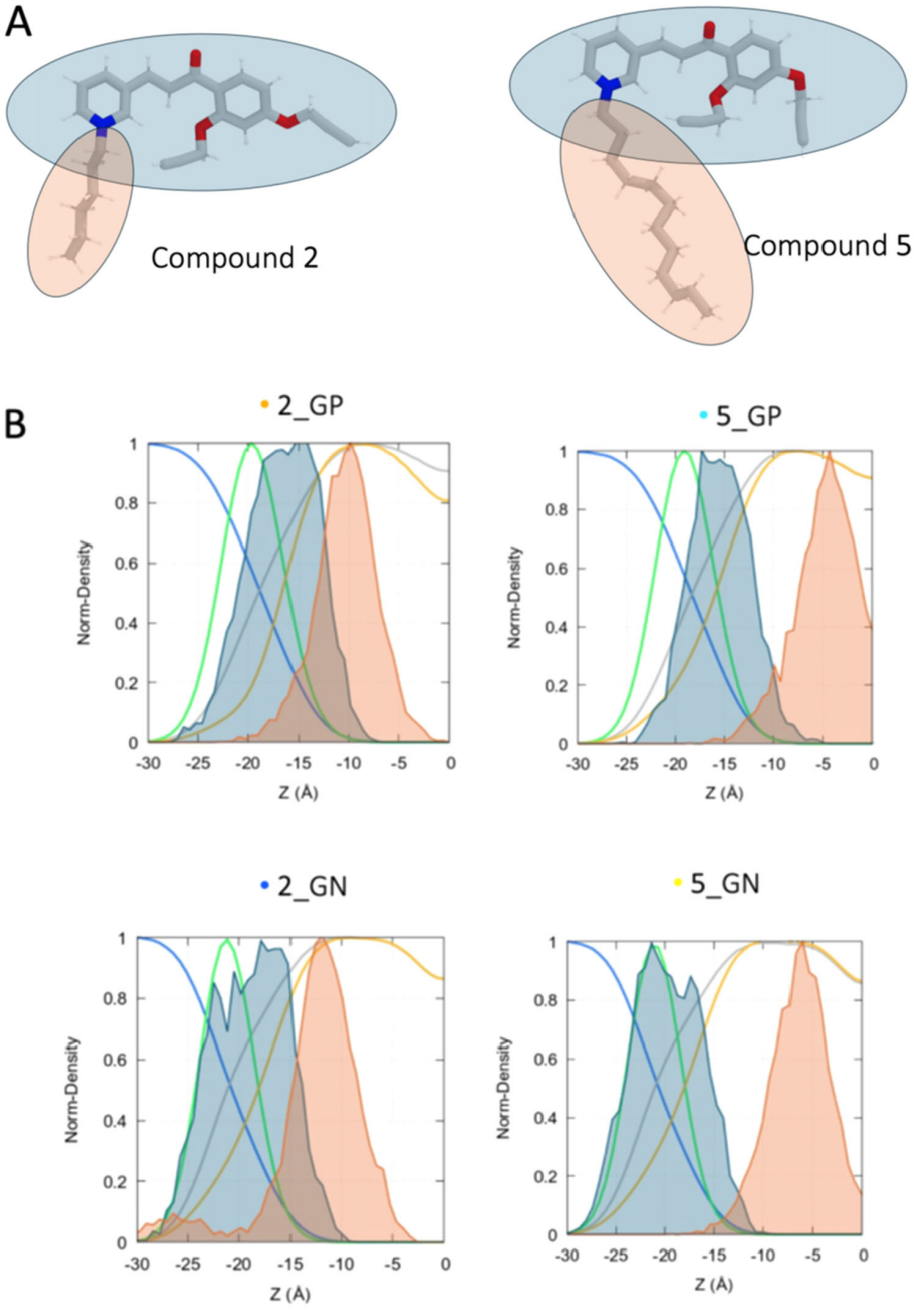
Ligand intercalation profiles. (A) Structural 3D representations of molecules **2** and **5**. Planar head and alkyl tail highlighted in light blue and light pink, respectively. (B) Mass density distribution profiles of the four simulated membranes with the intercalated surfactant. For simplicity, here are presented the distributions of the leaflet on which the QPySs are absorbed. The blue traces stand for the water molecules density profile, green for the lipid polar head, grey for the entire lipid, orange for the lipid tails, light blue for the QPyS polar head, and pink for the QPyS tail (consistently with the previously reported 3D molecular representations).

The mass density distribution profiles of the four simulated membranes with the intercalated QPyS **2** and **5** within membrane systems are provided by observing their mass density profiles projected along the membrane normal (*z*‐axis, Figure 6, Panel B). Regardless of the composition, the longer alkyl chain of **5** (highlighted in light pink) tended to penetrate deeper into the hydrophobic region of the membrane (represented by the orange and grey traces) compared with **2**. On the contrary, only the planar head (highlighted in light blue) of analog **5** showed to be positioned near the lipid polar head (green traces).

Additionally, the density profiles indicated that **2** and **5** appeared to intercalate deeper into the Gram‐positive membrane leaflet, with the polar heads positioned slightly above the membrane's polar edge. In contrast, interaction with Gram‐negative resulted in an overlap between the polar heads of lipids and the QPyS polar moiety.

**Figure 7:**
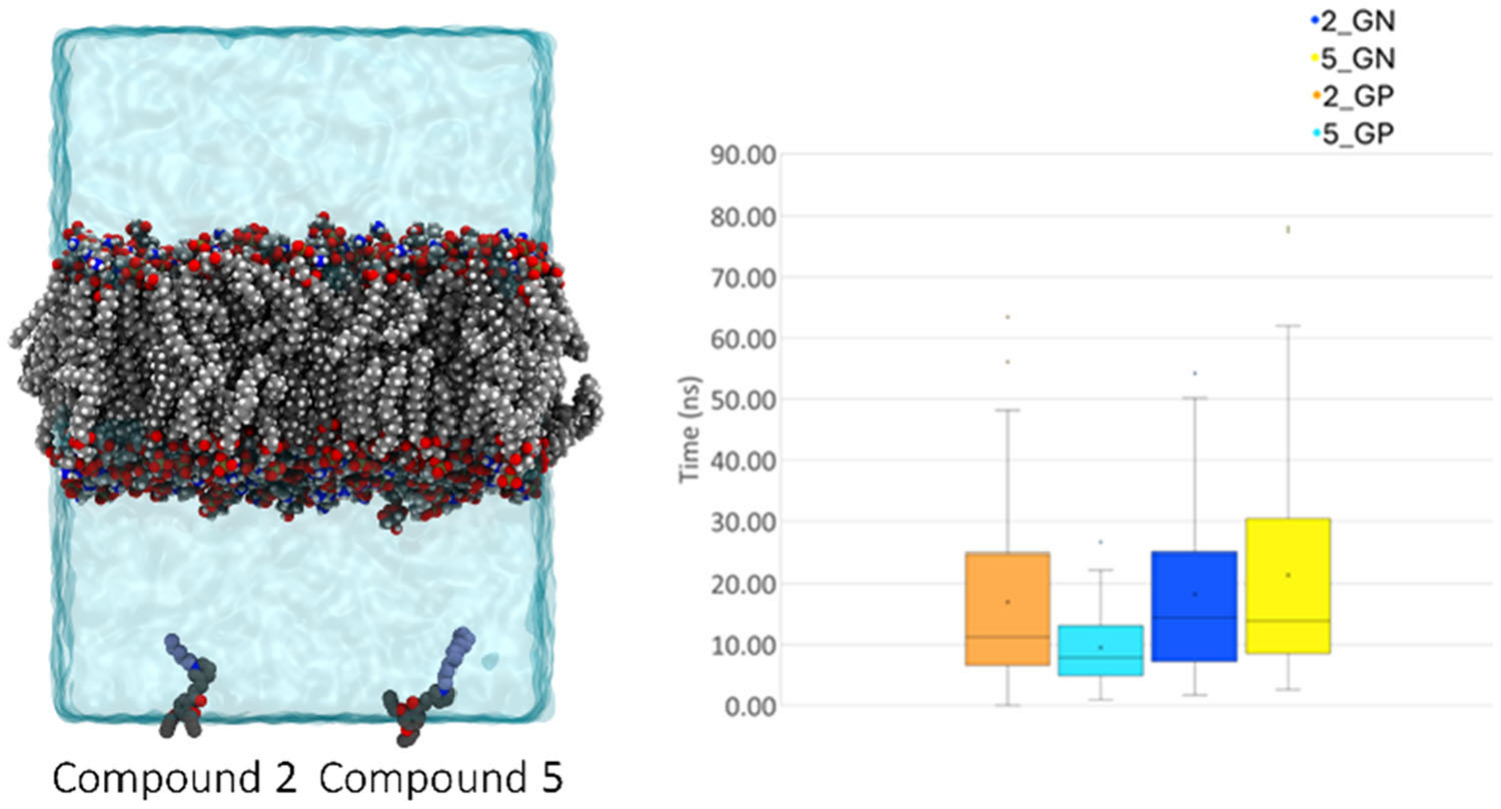
Simulation cell membrane representation with compounds **2** and **5** in bulk solution. The box plot shows the average diffusion time of ligands to the membrane surfaces.

Furthermore, the diffusion rates of the analogs were assessed during the transition of the QPyS from the bulk solution to the membrane leaflet. Derivatives **2** and **5** were simulated in the presence of both types of bacterial membranes, resulting in four diverse sets of 50 MD runs each. In each system, the QPySs diffused freely across periodic boundary conditions, potentially intercalating into the membrane from both leaflets. The measured times for each pool of simulations (**2**_GP, **5**_GP, **2**_GN, **5**_GN) were collected and averaged to establish a profile of absorption. In the GP model system, **5** exhibited the fastest absorption rate, spending an average of 9 ns (ns) in solution, while **2** showed significantly slower rates. On the other hand, in the GN model system, a similar absorption rate was observed (Figure 7, box plots: **2**_GP: 17 ns, **5**_GP: 9 ns, **2**_GN: 18 ns, and **5**_GN: 21 ns). Differences in membrane surface polarity, particularly in Gram‐positive membranes containing a higher concentration of negatively charged POPG lipids compared with neutral POPE lipids, may also influence the QPySs diffusion; in fact, both compounds displayed lower absorption rates in the almost negatively charged surface of Gram‐positive membranes. This effect was likely ascribed to an electrostatic attraction between the positively charged ligand and the double leaflet.

These findings highlight a complex interplay between molecular structure, membrane composition, and surface polarity, contributing to a deeper understanding of the mechanisms underlying QPySs' membrane interactions. The overall results could then explain the tendency of molecule **5** to show a higher efficiency in interacting with Gram‐positive membranes, suggesting a favorable activity related to its intercalation ability.

## 3 Conclusion

In the present study, a small series of QPySs (**2–10**) was designed and synthesized by properly decorating a pyridine‐based chalcone lead **1** as the head framework to obtain antibacterial and antifungal agents.

In particular, analogs **2–8** were characterized by a flexible linear alkyl function with lengths ranging from 6 to 18 carbon units (C6‐C18); moreover, **9** and **10** were designed to validate the involvement of the positively charged pyridinium substitution in the QPySs effects (as the central working hypothesis). Moreover, the simplified **11**, based on the 3‐methylpyridinium fragment, allowed to investigate the effect of the chalcone scaffold.

Several experimental settings were employed to evaluate the inhibitory properties of the new analogs **2–10**: first, the antimicrobial effect was assayed In Vitro against three priority human pathogens, *S. aureus*, *E. coli*, and *C. albicans*. These microorganisms were selected as representative strains for Gram‐positive and Gram‐negative bacteria and as fungal model systems. They are all causative agents of community‐ and hospital‐acquired infections, characterized by high recurrence rates and increasing antimicrobial resistance to many classes of antimicrobial agents.

Compounds evaluated against planktonic and biofilm forms of *S. aureus*, *E. coli*, and *C. albicans* revealed a common trend as the antimicrobial potency proved to be strictly related to the length of the alkyl tail. The optimal inhibitory effects on bacterial proliferation (low‐μM inhibition values) were observed for two derivatives (**4** and **5**, Table 1, Figure 2) bearing C10 and C12 side alkyl functions, respectively. In detail, analogs **3–7** effectively impacted *S. aureus* growth (MIC values ranging from 1.56 to 12.5 μM), while *E. coli* was inhibited only by **3–5** (MIC values ranging from 6.25 to 12.5 μM). Generally, a higher activity against *S. aureus* compared with *E. coli* was measured, possibly due to the reduced diffusion of the derivatives through the OM in Gram‐negative bacteria, which is a well‐known robust physicochemical defense for the cells. Indeed, when the compounds were assayed in combination with colistin, a conventional OM‐damaging antibiotic, the potency of **2** and **6** was restored. Regarding *C. albicans* proliferation, derivatives **4–7** turned out to be endowed with a remarkable antifungal efficacy (1.56–6.25 μM MIC values). Analogs **4** and **6**, displaying 1.56 μM values of MIC, emerged as the most effective inhibitors of the series against *S. aureus* and C*. albicans*, respectively. Among the tested compounds, analog **4** exhibited the highest activity against the *S*. *aureus* and *E. coli* biofilms; analog **6** displayed a strong antibiofilm efficacy against *C. albicans*.

Remarkably, the active analogs **4–6** were evaluated for their effects on both Vero cells and human erythrocytes and they proved to be safe at the inhibitory concentrations, supported by a lack of cytotoxicity as well as hemolytic effects, thus offering promises for a future clinical use.

To give a qualitative depiction of the QPySs's mode of action, along with corroborating the experimental results, MD simulations of the prototypical molecules **2** and **5**, with C6 and C12 tails, respectively, were performed by modeling them on Gram‐positive and Gram‐negative membranes. A framework to assess the antibacterial efficacy of the selected analogs in terms of diffusion rates was provided. Within the simulation, compound **5** proved to adopt an optimal l‐shaped conformation; it also exhibited the fastest absorption rate into the cell membranes and the highest efficiency to interact with Gram‐positive membranes, while compound **2** remained longer in the aqueous surroundings, thus supporting the experimentally observed differences in antimicrobial potencies. Overall, this computational methodology permits a deep understanding of the mechanistic insights of this QPyS class, and it also represents a useful tool for bioactivity prediction of newly designed analogs.

In summary, in the present study, analog **1**, characterized by a pyridine‐3‐yl chalcone framework, was purposely modified by inserting alkyl functions with lengths varying from 6 to 18 carbon units, thus obtaining a series of QPyS. Among the developed compounds, some interesting leads were identified as being endowed with consistent antibacterial and antifungal activities. In particular, analogs **4–6** proved to be active toward *S. aureus*, *E. coli*, and *C. albicans*. Interestingly, for the best‐performing analog **5**, the ability to target microbial membranes was confirmed as a peculiar mechanism of action by employing computational studies. In summary, the insights presented here can be used to guide the further optimization of the emerged lead compounds toward the development of new classes or prototypes of antimicrobial candidates.

## 4 Experimental

### 4.1 Chemistry

#### 4.1.1 General Chemistry Information

Chemical reagents and solvents, unless otherwise specified, were employed as commercial products with a high‐grade purity. Reaction courses were monitored by thin‐layer chromatography (TLC) performed on precoated TLC plates (Merck Silica Gel 60 F254, layer 0.20 mm) and then visualized under a UV lamp (λ = 254 and 365 nm). Chromatographic separations were performed on silica gel columns using the flash method (Flash column chromatography, FCC, Kiesegel 40, particle size 0.040–0.063 mm, Merck). Melting points were determined in open glass capillaries using a Büchi apparatus and were uncorrected. ^1^H NMR and ^13^C NMR spectra were recorded on a Varian INOVA spectrometer operating at 400 and 101 MHz, and on a Bruker spectrometer working at 600 and 150 MHz, respectively; data are reported as follows: chemical shift (ppm δ value), multiplicity (indicated as: br., broad signal; s, singlet; d, doublet; t, triplet; q, quartet; p, quintet; m, multiplet and combinations thereof), coupling constants (*J*) in Hertz (Hz) and integrated intensity. HRMS spectra were recorded on a Waters Xevo G2‐XS quadrupole time‐of‐flight apparatus operating in electrospray mode. UHPLC − MS analyses were run on a Waters ACQUITY ARC UHPLC/MS system consisting of a QDA mass spectrometer equipped with an electrospray ionization interface and a 2489 UV/Vis detector; the detected wavelength was 365 nm. Analyses were performed on an XBridge BEH C18 column (10 × 2.1 mm i.d., particle size 2.5 μm) with an XBridge BEH C18 VanGuard Cartridge precolumn (5 mm × 2.1 mm i.d., particle size 1.8 μm); the mobile phases were H_2_O (0.1% formic acid) (A) and MeCN (0.1% formic acid) (B). Linear gradient: 0−0.78 min, 20% B; 0.78–2.87 min, 20%−95% B; 2.87–3.54 min, 95% B; 3.54–3.65 min, 95%–20% B; 3.65–5.73, 20% B. Flow rate: 0.8 mL/min. Electrospray ionization in positive and negative modes was applied in the mass scan range of 50−1200 Da. All tested compounds were found to have > 95% purity. The compound's name is in accord with the naming algorithm developed by CambridgeSoft Corporation and used in Chem‐BioDraw Ultra 22.2.0.

The InChI codes of the investigated compounds, together with some biological activity data, are provided as Supporting Information.

#### 4.1.2 General Procedure for the Synthesis of QPySs 2–10 (Menshutkin‐Type Reaction)

To a solution of compound **1** [24] (1.0 eq) in acetonitrile (10 mL/eq) at room temperature, the corresponding alkyl halide (3.0 eq) was added. The resulting reaction mixture was stirred at 80°C for 24–48 h, and the reaction progress was monitored by TLC. The solvent was evaporated under reduced pressure, and the resulting residue was treated with *n*‐hexane (10 mL/eq) to remove the unreacted alkyl halide. The obtained solid was recovered by filtration and purified by FCC (gradient elution: ethyl acetate, then ethyl acetate/methanol), yielding the desired product.

(*E*)‐3‐{3‐[2,4‐Bis(prop‐2‐yn‐1‐yloxy)phenyl]‐3‐oxoprop‐1‐en‐1‐yl}‐1‐hexylpyridin‐1‐ium bromide (**2**): Starting from **1** (0.23 g, 0.73 mmol), 1‐Br‐hexane (0.36 g, 0.30 mL, 2.19 mmol) in acetonitrile (7.3 mL) and following the General Procedure (reaction time 24 h), a crude product was obtained that was purified by FCC (ethyl acetate/methanol 9:1; Rf = 0.3) affording **2** as a solid (0.18 g, 51% yield), mp: 104°C–105°C. ^1^H NMR (600 MHz, DMSO‐*d*_*6*_) δ (ppm) 9.45 (d, *J* = 1.6 Hz, 2H, H‐2’), 9.07 (d, *J* = 6.0 Hz, 1H, H‐4’), 8.92 (d, *J* = 8.2 Hz, 1H, H‐6’), 8.21 (dd, *J* = 8.2, 6.0 Hz, 1H, H‐5’), 7.88 (d, *J* = 16.0 Hz, 1H, α‐C=CH), 7.70 (d, *J* = 8.6 Hz, 1H, H‐6), 7.60 (d, *J* = 16.0 Hz, 1H, β‐C=CH), 6.87 (d, *J* = 2.2 Hz, 1H, H‐3), 6.80 (dd, *J* = 8.6, 2.2 Hz, 1H, H‐5), 4.97 (d, *J* = 2.4 Hz, 2H, OCH_2_), 4.93 (d, *J* = 2.4 Hz, 2H, OCH_2_), 4.61–4.58 (m, 2H, CH_2_N^+^), 3.65 (t, *J* = 2.4 Hz, 1H, C≡CH), 3.62 (t, *J* = 2.4 Hz, 1H, C≡CH), 1.90–2.00 (m, 2H, CH_2_), 1.34–1.24 (m, 6H, (CH_2_)_3_), 0.89‐0.79 (m, 3H, CH_3_). ^13^C NMR (151 MHz, DMSO‐*d*_6_) δ (ppm) 188.61 C═O), 162.14 (C4), 158.37 C2), 144.65 (C2‘), 144.59 (C4‘), 142.65 (C6‘), 135.32 (C1‘), 133.47 (Cα), 133.08 (Cβ), 132.20 (C6), 128.04 (C5‘), 121.60 (C1), 107.56 (C3), 101.36 C5), 79.22 (C≡), 79.00 (C≡), 78.78 (≡C), 78.58 (≡C), 61.16 (C1“), 56.73 (OCH_2_), 56.02 (OCH_2_), 30.59 (C2“), 30.44 25.09, 21.86 (C3“‐C5“), 13.87 (CH_3_). HRMS (*m/z*)*:* [M+H]^+^ calc for C_26_H_29_BrNO_3_ 482.1331; found 482.1330.

(*E*)‐3‐{3‐[2,4‐Bis(prop‐2‐yn‐1‐yloxy)phenyl]‐3‐oxoprop‐1‐en‐1‐yl}‐1‐octylpyridin‐1‐ium bromide (**3**): Starting from **1** (0.23 g, 0.73 mmol), 1‐Br‐octane (0.42 g, 0.38 mL, 2.19 mmol) in acetonitrile (7.3 mL) and following the General Procedure (reaction time 30 h), a crude product was obtained that was purified by FCC (ethyl acetate/methanol 9:1, Rf = 0.3) affording **3** as a solid (0.10 g, 27% yield), mp: 107°C–109°C. ^1^H NMR (600 MHz, DMSO‐*d*_6_) *δ* (ppm) 9.49 (t, 1H, *J* = 1.7 Hz, H‐2’), 9.10 (dd, *J* = 6.0, 1.3 Hz, 1H, H‐4’), 8.94 (dt, *J* = 8.2, 1.5 Hz, 1H, H‐6’), 8.22 (dd, *J* = 8.2, 6.0 Hz, 1H, H‐5’), 7.89 (d, *J* = 16.0 Hz, 1H, α‐C═CH), 7.70 (d, *J* = 8.6 Hz, 1H, H‐6), 7.60 (d, *J* = 16.0 Hz, 1H, β‐C═CH), 6.87 (d, *J* = 2.2 Hz, 1H, H‐3), 6.80 (dd, *J* = 8.6, 2.2 Hz, 1H, H‐5), 4.98 (d, *J* = 2.4 Hz, 2H, OCH_2_), 4.94 (d, *J* = 2.4 Hz, 2H, OCH_2_), 4.60 (t, *J* = 7.6 Hz, 2H, CH_2_N^+^), 3.66 (t, *J* = 2.4 Hz, 1H, C≡CH), 3.63 (d, *J* = 2.4 Hz, 1H, C≡CH), 2.01–1.93 (m, 2H, CH_2_CH_2_N^+^), 1.33–1.24 (m, 10 H, (CH_2_)_5_), 0.85 (t, *J* = 6.6 Hz, 3H, CH_3_); ^13^C NMR (151 MHz, DMSO‐*d*_6_) *δ* (ppm) 189.05 (C═O), 162.59 (C4), 158.83 (C2), 145.11 (C2‘), 145.04 (C4‘), 143.11 (C6‘), 135.75 (C1‘), 133.93 (Cα), 133.52 (Cβ), 132.65 (C6), 128.49 (C5‘), 122.05 (C1), 108.02 (C3), 101.81 (C5), 79.43 (C≡), 79.38 (C≡), 79.18 (≡C), 79.03 (≡C), 61.60 (C1“), 57.19 (OCH_2_), 56.48 (OCH_2_), 31.63 (C2“), 30.95 28.90, 28.85, 25.89, 22.53 (C3“‐C7“), 14.43 (CH_3_). HRMS (*m/z*)*:* [M+H]^+^ calc for C_28_H_33_BrNO_3_ 510.1644; found 510.1640.

(*E*)‐3‐{3‐[2,4‐Bis(prop‐2‐yn‐1‐yloxy)phenyl]‐3‐oxoprop‐1‐en‐1‐yl}‐1‐decylpyridin‐1‐ium bromide (**4**): Starting from **1** (0.15 g, 0.47 mmol), 1‐Br‐decane (0.31 g, 0.29 mL, 1.41 mmol), acetonitrile (4.7 mL) and following the General Procedure (reaction time 32 h) a crude product was obtained that was purified by FCC (ethyl acetate/methanol 9:1¸ Rf = 0.3) affording **4** as a solid (0.09 g, 35% yield), mp:131°C–133°C. ^1^H NMR (600 MHz, CDCl_3_) *δ* (ppm) 9.64 (s, 1H, H‐2’), 9.35 (d, *J* = 6.0 Hz, 1H, H‐4’), 8.57 (d, *J* = 8.2 Hz, 1H, H‐6’), 8.17–8.13 (m, 2H, αC=CH and CH‐5’), 7.92 (d, *J* = 8.5 Hz, 1H, H‐6), 7.58 (d, *J* = 15.8 Hz, 1H, βC=CH), 6.73–6.70 (m, 2H, H‐5 and H‐3), 5.09 (t, *J* = 7.6 Hz, 2H, CH_2_N^+^), 4.97 (d, *J* = 2.4 Hz, 2H, OCH_2_), 4.78 (d, *J* = 2.4 Hz, 2H, OCH_2_), 2.68 (t, *J* = 2.4 Hz, 1H, C≡CH), 2.59 (t, *J* = 2.4 Hz, 1H, C≡CH), 2.06 (q, *J* = 6.1 Hz, 2H, CH_2_CH_2_N^+^), 1.43–1.32 (m, 14H, (CH_2_)_7_), 0.87 (t, *J* = 7.0 Hz, 3H, CH_3_). ^13^C NMR (151 MHz, CDCl_3_) *δ* (ppm) 187.68 05 (C═O), 162.83 (C4), 158.98 (C2), 144.26 (C2‘), 143.46 (C4‘), 142.72 (C6‘), 136.57 (C1‘), 134.64(Cα), 133.51 (Cβ), 131.68 (C6), 128.38(C5‘), 121.48 (C1), 107.57 (C3), 100.67 (C5), 77.93 (C≡), 77.47 (C≡), 76.80 (≡C), 76.53 (≡C), 57.11 (OCH_2_), 56.08 (OCH_2_), 31.94, 31.58, 28.94, 28.91, 26.00, 25.53, 22.49 (C3“‐C9“), 13.98 (CH_3_). HRMS (*m/z*)*:* [M+H]^+^ calc for C_30_H_37_BrNO_3_ 538.1957 found 538.1970.

(*E*)‐3‐{3‐[2,4‐Bis(prop‐2‐yn‐1‐yloxy)phenyl]‐3‐oxoprop‐1‐en‐1‐yl}‐1‐dodecylpyridin‐1‐ium bromide (**5**): Starting from **1** (0.20 g, 0.63 mmol), 1‐Br‐dodecane (0.20 g, 0.27 mL, 1.9 mmol) in acetonitrile (6.3 mL) and following the General Procedure (reaction time 30 h), a crude product was obtained that was purified by FCC (ethyl acetate/methanol 9:1¸ Rf = 0.3) affording **5** as a solid (0.09 g, 25% yield), mp:130°C–132°C. ^1^H NMR (400 MHz, CDCl_3_) *δ* (ppm) 9.45 (s, 1H, H‐2’), 9.07 (d, *J* = 6.0 Hz, 1H, H‐4’), 8.92 (d, *J* = 8.4 Hz, 1H, H‐6’), 8.23–8.19 (m, 1H, H‐5’), 7.88 (d, *J* = 16.0 Hz, 1H, αC=CH), 7.70 (d, *J* = 8.8 Hz, 1H, H‐6), 7.59 (d, *J* = 16.0 Hz, 1H, βC=CH), 6.87 (d, *J* = 2.4 Hz, 1H, H‐3), 6.80 (dd, *J* = 2.0, 8.8 Hz, 1H, H‐5), 4.97 (d, *J* = 2.4 Hz, 2H, OCH_2_), 4.03 (d, *J* = 2.0 Hz, 2H, OCH_2_), 4.58 (t, *J* = 7.4, 2H, CH_2_N^+^), 3.62–3.52 (m, 2H, CH_2_), 2.01–1.95 (m, 2H, C≡CH), 1.34–1.18 (m, 18H, (CH_2_)_9_), 0.85 (t, *J* = 6.4 Hz, 3H, CH_3_). ^13^C NMR (101 MHz, CDCl_3_) *δ* (ppm) 187.73 (C═O),162.96 (C4), 159.10 (C2), 144.24 (C2‘), 143.36 (C4‘), 142.78 (C6‘), 136.87 (C1‘), 135.02 (Cα), 133.65 (Cβ), 131.53 (C6), 128.36 (C5‘), 121.57 (C1), 107.66 (C3), 100.76 (C5), 78.02 (C≡), 77.53 (C≡), 65.87 (≡C), 62.57 (≡C), 57.20 (OCH_2_), 56.14 (OCH_2_), 32.03 1 (C2“), 31.92, 29.67, 29.64, 29.62, 29.58, 29.49, 29.36, 29.33, 29.07, 26.10, 22.69 (C3“‐C11“), 14.14 (CH_3_). HRMS (*m/z*)*:* [M+H]^+^ calc for C_32_H_41_BrNO_3_ 566.2270; found 566.2278.

(*E*)‐3‐{3‐[2,4‐Bis(prop‐2‐yn‐1‐yloxy)phenyl]‐3‐oxoprop‐1‐en‐1‐yl}‐1‐tetradecylpyridin‐1‐ium bromide (**6**): Starting from **1** (0.16 g, 0.5 mmol), 1‐Br‐tetradecane (0.415 g, 0.44 ml, 1.5 mmol) in acetonitrile (5 mL) and following the General Procedure (reaction time 36 h), a crude product was obtained that was purified by FCC (ethyl acetate/methanol 9:1¸ Rf = 0.3) affording **6** as a solid (0.02 g, 7% yield), mp: 109°C–111°C. ^1^H NMR (600 MHz, DMSO‐*d*_6_) *δ* (ppm) 9.45 (t, 1H, *J* = 1.7 Hz, H‐2’), 9.04 (dd, *J* = 6.0, 1.3 Hz, 1H, H‐4’), 8.92 (dd, *J* = 8.2, 1.5 Hz, 1H, H‐6’), 8.21 (dd, *J* = 8.2, 6.0 Hz, 1H, H‐5’), 7.82 (d, *J* = 16.0 Hz, 1H, α‐C=CH), 7.70 (d, *J* = 8.6 Hz, 1H, H‐6), 7.56 (d, *J* = 16.0 Hz, 1H, β‐C=CH), 6.87 (d, *J* = 2.2 Hz, 1H, H‐3), 6.80 (dd, *J* = 8.6, 2.2 Hz, 1H, H‐5), 4.97 (d, *J* = 2.4 Hz, 2H, OCH_2_), 4.93 (d, *J* = 2.4 Hz, 2H, OCH_2_), 4.57 (t, *J* = 7.6 Hz, 2H, CH_2_N^+^), 3.68 (t, *J* = 2.4 Hz, 1H, C≡CH), 3.62 (d, *J* = 2.4 Hz, 1H, C≡CH), 1.95 (t, *J* = 7.4 Hz, CH_2_CH_2_N^+^), 1.29–1.21 (m, 22 H, (CH_2_)_11_), 0.84 (t, *J* = 6.6 Hz, 3H, CH_3_). ^13^C NMR (151 MHz, DMSO‐*d*_6_) δ (ppm) 188.73 (C═O), 162.28 (C4), 158.5 (C2), 144.79 (C2‘), 144.73(C4‘), 142.77 (C6‘), 135.45 (C1‘), 133.2 (Cα), 133.62 (Cβ), 132.34 (C6), 128.17 (C5‘), 121.74 (C1), 107.69 (C3), 101.50 (C5), 79.11 (C≡), 79.07 (C≡), 78.87 (≡C), 78.73 (≡C), 61.32 (C1“), 56.86 (OCH_2_), 56.16 (OCH_2_), 31.45 (C2“), 30.59, 29.22, 29.20, 29.17, 29.08, 28.91, 28.87, 28.55, 25.55, 22.26 (C3“‐C13“), 14.14 (CH_3_). HRMS (ESI) (*m/z*)*:* [M+H]^+^ calc for C_34_H_45_BrNO_3_ 594.2583; found 594.2578.

(*E*)‐3‐{3‐[2,4‐Bis(prop‐2‐yn‐1‐yloxy)phenyl]‐3‐oxoprop‐1‐en‐1‐yl}‐1‐hexadecylpyridin‐1‐ium (**7**): Starting from **1** (0.19 g; 0.6 mmol), 1‐Br‐hexadecane (0.55 g, 0.55 mL, 1.8 mmol) in acetonitrile (6 mL) and following the General Procedure (reaction time 36 h), a crude product was obtained that was purified by FCC (ethyl acetate/methanol 9:1¸ Rf = 0.35) affording **7** as a solid (0.06 g, 16% yield), mp: 112°C–114°C. ^1^H NMR (600 MHz, CDCl_3_) *δ* (ppm) 9.61 (s, 1H, H‐2’), 9.33 (d, *J* = 6.0 Hz, 1H, H‐4’), 8.58‐8.56 (m, 1H, H‐6’), 8.14 (dd, *J* = 8.4, 6.0 Hz, 1H, H‐5’), 8.09 (d, *J* = 15.8 Hz, 1H, αC=CH), 7.91 (d, *J* = 8.8 Hz, 1H, H‐6), 7.57 (d, *J* = 15.8 Hz, 1H, βC=CH), 6.72‐6.70 (m, 2H, H‐3 and H‐5), 5.09 (t, *J* = 7.5 Hz, 2H, N^+^CH_2_), 4.97 (d, *J* = 2.4 Hz, 2H, OCH_2_), 4.77 (d, *J* = 2.4 Hz, 2H, OCH_2_), 2.69 (t, *J* = 2.4 Hz, 1H, C≡CH), 2.59 (t, *J* = 2.4 Hz, 1H, C≡CH), 2.09–2.02 (m, 2H, CH_2_), 1.47–1.18 (m, 26H, (CH_2_)_13_), 0.87 (t, *J* = 6.8 Hz, 3H, CH_3_). ^13^C NMR (151 MHz, CDCl_3_) δ 187.74 (C = O), 162.95 (C4), 159.09 (C2), 144.27 (C2‘), 143.39 (C4‘), 142.76 (C6‘), 136.81 (C1‘), 134.92 (Cα), 133.64 (Cβ), 131.6 (C6), 128.4 (C5‘), 121.56 (C1), 107.66 (C3), 100.75 (C5), 78.01 (C≡), 77.57 (C≡), 77.23 (≡C), 77.11 (≡C), 57.18 (OCH_2_), 56.15 (OCH_2_), 32.01, 31.93, 30.96, 29.71, 29.66, 29.61, 29.59, 29.51, 29.37, 29.34, 29.08, 26.10, 22.70 (C3“‐C15“), 14.14 (CH_3_). HRMS (ESI) (*m/z*)*:* [M+H]^+^ calc for C_34_H_45_BrNO_3_ *m/z:* calcd 656.2506 found 656.2518.

(*E*)‐3‐{3‐[2,4‐Bis(prop‐2‐yn‐1‐yloxy)phenyl]‐3‐oxoprop‐1‐en‐1‐yl}‐1‐octadecylpyridin‐1‐ium bromide (**8**). Starting from **1** (0.17 g, 0.54 mmol), 1‐Br‐octadecane (0.53 g, 0.54 mL, 1.59 mmol) in acetonitrile (5.4 mL) and following the General Procedure (reaction time 40 h), a crude product was obtained that was purified by FCC (ethyl acetate/methanol 4:1¸ Rf = 0.2) affording **8** as a solid (0.09 g, 25% yield), mp:148°C–149°C. ^1^H NMR (600 MHz, DMSO‐*d*_6_) *δ* (ppm) 9.47 (t, 1H, *J* = 1.7 Hz, H‐2’), 9.09 (dd, *J* = 6.0, 1.3 Hz, 1H, H‐4’), 8.82 (dt, *J* = 8.2, 1.5 Hz, 1H, H‐6’), 8.21 (dd, *J* = 8.2, 6.0 Hz, 1H, H‐5’), 7.89 (d, *J* = 16.0 Hz, 1H, α‐C=CH), 7.70 (d, *J* = 8.6 Hz, 1H, H‐6), 7.59 (d, *J* = 16.0 Hz, 1H, β‐C=CH), 6.87 (d, *J* = 2.2 Hz, 1H, H‐3), 6.79 (dd, *J* = 8.7, 2.3 Hz, 1H, H‐5), 4.97 (d, *J* = 2.4 Hz, 2H, OCH_2_), 4.93 (d, *J* = 2.4 Hz, 2H, OCH_2_), 4.58 (t, *J* = 7.6 Hz, 2H, CH_2_N^+^), 3.66 (t, *J* = 2.4 Hz, 1H, C≡CH), 3.63 (d, *J* = 2.4 Hz, 1H, C≡CH), 1.97‐1.94 (m, 2H, CH_2_CH_2_N^+^), 1.33–1.17 (m, 30 H, (CH_2_)_15_), 0.85 (t, *J* = 6.6 Hz, 3H, CH_3_). ^13^C NMR (151 MHz, DMSO‐*d*_6_) δ (ppm) 188.73 (C═O), 162.29 (C4), 158.52 (C2), 144.81 (C2‘), 144.74 (C4‘), 142.79 (C6‘), 135.44 (C1‘), 133.62 (Cα), 133.21 (Cβ), 132.35 (C6), 128.18 (C5‘), 121.73 (C1), 107.7 (C3), 101.5 (C5), 79.12 (C≡), 79.08 (C≡), 78.88 (≡C), 78.73 (≡C), 61.3 (C1“), 56.88 (OCH_2_), 56.17 (OCH_2_), 31.46 (C2“), 30.61, 29.22, 29.17, 29.13, 29.09, 28.95, 28.92, 28.87, 28.56, 25.56, 22.27 C3“‐C17“), 14.13 (CH_3_). HRMS (ESI) (*m/z*)*:* [M+H]^+^ calcd for C_38_H_53_BrNO_3_ 650.3209; found 650.3207.

(*E*)‐3‐{3‐[2,4‐Bis(prop‐2‐yn‐1‐yloxy)phenyl]‐3‐oxoprop‐1‐en‐1‐yl}pyridin‐1‐ium bromide (**9**): Starting from **1** (0.17 g, 0.54 mmol), 48% HBr (0.25 g, 0.17 mL, 1.59 mmol) in acetonitrile (5.4 mL) and following the General Procedure (reaction time 40 h), a crude product was obtained that was purified by FCC (ethyl acetate/methanol 4:1¸ Rf = 0.2) affording **9** as a solid (0.15 g, 70% yield), mp: dec. 180°C. ^1^H NMR (600 MHz, DMSO‐*d*_6_) *δ* (ppm) 9.47 (t, 1H, *J* = 1.7 Hz, H‐2’), 9.09 (dd, *J* = 6.0, 1.3 Hz, 1H, H‐4’), 8.82 (dd, *J* = 8.2, 1.5 Hz, 1H, H‐6’), 8.21 (dd, *J* = 8.2, 6.0 Hz, 1H, H‐5’), 7.89 (d, *J* = 16.0 Hz, 1H, α‐C=CH), 7.70 (d, *J* = 8.6 Hz, 1H, H‐6), 7.59 (d, *J* = 16.0 Hz, 1H, β‐C=CH), 6.87 (d, *J* = 2.2 Hz, 1H, H‐3), 6.79 (dd, *J* = 8.7, 2.3 Hz, 1H, H‐5), 4.97 (d, *J* = 2.4 Hz, 2H, OCH_2_), 4.93 (d, *J* = 2.4 Hz, 2H, OCH_2_), 4.58 (t, *J* = 7.6 Hz, 2H, CH_2_N^+^), 3.66 (t, *J* = 2.4 Hz, 1H, C≡CH), 3.63 (d, *J* = 2.4 Hz, 1H, C≡CH), 1.97–1.94 (m, 2H, CH_2_CH_2_N^+^), 1.33–1.17 (m, 30 H, (CH_2_)_15_), 0.85 (t, *J* = 6.6 Hz, 3H, CH_3_).^13^C NMR (151 MHz, DMSO‐*d*_6_) δ(ppm) 188.73 (C═O), 162.29 (C4), 158.52 (C2), 144.81 (C2‘), 144.74 (C4‘), 142.79 (C6‘), 135.44 (C1‘), 133.62 (Cα), 133.21 (Cβ), 132.35 (C6), 128.18 (C5‘), 121.73 (C1), 107.7 (C3), 101.5 (C5), 79.12 (C≡), 79.08 (C≡), 78.88 (≡C), 78.73 (≡C), 61.3 (C1“), 56.88 (OCH_2_), 56.17 (OCH_2_), 31.46 (C2“), 30.61, 29.22, 29.17, 29.13, 29.09, 28.95, 28.92, 28.87, 28.56, 25.56, 22.27 (C3“‐C17“), 14.13 (CH_3_). HRMS (ESI) (*m/z*) [M+H]^+^ calc for C_20_H_17_BrNO_3_ 398.0392; found 398.0398.

1,1′‐(Hexane‐1,6‐diyl)bis{3‐[(*E*)‐3‐(2,4‐bis‐prop‐2‐yn‐1‐yloxyphenyl)‐3‐oxoprop‐1‐en‐1‐yl]pyridin‐1‐ium} bromide (**10**): Starting from **1** (0.23 g, 0.73 mmol), 1,6‐di‐Br‐hexane (1.07 g, 0.67 mL, 4.38 mmol) in acetonitrile (7.3 mL) and following the General Procedure (reaction time 48 h), a crude product was obtained that was purified by FCC (ethyl acetate/methanol 9:1, Rf = 0.2) affording **10** as a solid (0.45 g, 68% yield), mp: 161°C–1612°C. ^1^H NMR (600 MHz, CDCl_3_) *δ* (ppm) 9.77 (s, 2H, H‐2’), 9.41 (d, *J* = 8.0 Hz, 2H, H‐4’), 8.59 (d, *J* = 8.1 Hz, 2H, H‐6’), 8.16–8.13 (m, 2H, H‐5’), 8.08 (d, *J* = 15.8 Hz, 2H, α‐C=CH), 7.89 (d, *J* = 8.4 Hz, 2H, H‐6), 7.58 (d, *J* = 15.8 Hz, 1H, β‐C=CH), 6.72–6.68 (m, 4H, H‐3, H‐5), 5.12 (t, *J* = 7.6 Hz, 4H, CH_2_N^+^), 4.96 (d, *J* = 2.4 Hz, 4H, OCH_2_), 4.77 (d, *J* = 2.4 Hz, 4H, OCH_2_), 3.40 (t, *J* = 6.6 Hz, 4H, CH_2_CH_2_N^+^), 2.7 (t, *J* = 2.4 Hz, 2H, C≡CH), 2.6 (t, *J* = 2.4 Hz, 2H, C≡CH), 2.11 (t, *J* = 7.1 Hz, 4H, (CH_2_)_2_).^13^C NMR (151 MHz, CDCl_3_) δ (ppm) 187.86 (C=O), 163.08 (C4), 159.2 (C2), 144.44 (C2‘), 143.74 (C4‘), 142.96 (C6‘), 136.98 (C1‘), 134.97 (Cα), 133.76 (Cβ), 131.82 (C6), 128.456 (C5‘), 121.68 (C1), 107.81 (C3), 100.90 (C5), 78.16 (C≡), 77.67 (C≡), 77.137 (≡C), 76.96 (≡C), 62.20 (C1“), 57.32 (OCH_2_), 56.29 (OCH_2_), 33.86 (C2“), 32.28, 32.01, 27.57, 25.28 (C3“‐C6“). HRMS (ESI) (*m/z*) [M+H]^+^ calc for C_46_H_43_Br_2_N_2_O_6_ 877.1488; found 877.1480.

1‐Hexadecyl‐3‐methylpyridin‐1‐ium bromide (**11**): Starting from 3‐methylpyridine (0.28 g, 3.0 mmol), 1‐Br‐hexadecane (0.91 g; 0.91 mL, 6.0 mmol) in acetonitrile (30 mL) and following the General Procedure (reaction time 24 h), a crude product was obtained that was purified by FCC (ethyl acetate/methanol 9.5:0.5; Rf = 0.2) affording **11** as a white solid (0.6 g, 55% yield), mp:109°C–111°C. ^1^H NMR (600 MHz, CDCl_3_) *δ* (ppm) 9.33 (s, 1H, H‐2), 9.21 (d, *J* = 5.9 Hz, 1H, H‐6), 8.23 (dt, *J* = 8.0, 1.2 Hz, 1H, H‐4), 7.98 (dd, *J* = 8.0, 6.0 Hz, 1H, H‐5), 4.94 (t, *J* = 7.5 Hz, 2H, CH_2_ N^+^), 2.64 (s, 3H, ArCH_3_), 2.02 (t, *J* = 7.6 Hz, 2H, CH_2_), 1.39‐1.24 (m, 22H, (CH_2_)_11_), 0.87 (t, *J* = 7.0 Hz, 3H, CH_3_). ^3^C NMR (151 MHz, CDCl_3_) δ (ppm) 145.66, 144.45, 142.23, 139.71, 127.81, 77.28, 77.07, 76.86, 62.07, 31.98, 31.94, 29.70, 29.66, 29.62, 29.54, 29.38, 29.11, 26.14, 22.71, 18.83, 14.16. HRMS (ESI) (*m/z*) [M+H]^+^ calc for C_20_H_37_BrNO 370.2109; found 370.2114.

### 4.2 Biological Assays

#### 4.2.1 Microbial Strains and Growth Conditions

Reference strains of *Staphylococcus aureus* (ATCC 25923), *Escherichia coli* (ATCC 25922), and *Candida albicans* (ATCC 10231) were used in this study as microbial models. These strains were purchased from the American Type Culture Collection (ATCC); bacterial cultures were routinely grown on 5% blood agar plate, while fungal cultures on Sabouraud Dextrose Agar (Biolife Italiana S.r.l., Milan, Italy).

#### 4.2.2 Chalcone‐Based QPySs and Reference Controls

The dry powder of the chalcone‐based QPySs was resuspended in dimethylsulfoxide (DMSO) at 20 mM and used as stock solutions. For biological investigations, compounds were used in the range 100–0.78 µM, and the solvent in the corresponding percentage range. The following commercially available antimicrobials and toxic clinical drugs were purchased from Sigma‐Aldrich (St. Louis, MO, USA), dissolved in water, and used as reference controls. Gentamicin and ampicillin were used in the range 107.69–0.82 µM and 143.23–1.12 µM, respectively; the colistin was used at 0.03 µM in association with the chalcone‐based QASs with *E. coli*. The fluconazole was used in the assays with *C. albicans* in the range 3.27–0.03 µM. The doxorubicin was included as a clinical drug control in the cytotoxicity studies with Vero cells (ATCC CCL‐81) in the range 367.97–0.72 µM.

#### 4.2.3 Determination of the MIC and IC_50_ Values

The antimicrobial activity of the compounds was assessed by determining the Minimum Inhibitory Concentration (MIC) by a well‐established broth microdilution procedure in microtiter plates and in compliance with the Clinical and Laboratory Standards Institute (CLSI) guidelines [33].

In short, microbial inocula were prepared at 0.5 McFarland in PBS and, subsequently, bacterial suspensions were diluted 1:200 in Mueller–Hinton broth (Sigma‐Aldrich, St. Louis, MO, USA), while fungal inoculum was diluted 1:20 in RPMI‐1640 medium (Gibco®, ThermoFisher Scientific Inc., Waltham, MA, USA), containing glucose 2%, 0.3% levo‐glutamine buffered to pH 7.0 with 0.165 M 3‐(N‐morpholino) propanesulfonic acid (MOPS). A total of 100 µL of these microbial suspensions was introduced into a 96‐well microplate and incubated with 100 µL of the compounds, two‐fold serially diluted in the range of 100–0.19 µM. Positive controls (microbial suspensions in regular media), negative controls (only compounds), and solvent controls (microbial suspensions incubated with DMSO dilutions) were included in the tests. The plate was incubated at 37°C for 24 h, and subsequently the optical density at 630 nm (OD_630nm_) was spectrophotometrically measured. Experiments were carried out in triplicate in three biological replicates. In addition to MIC, IC_50_ values were measured for the most active compounds. The values, corresponding to the concentration of the compound giving rise to an inhibition of growth of 50%, were obtained by interpolation on dose–response curves generated by plotting the percentages of growth inhibition, relative to the positive control (set to 100% of growth), as a function of the tested concentrations (on a logarithm scale). Statistical analysis was carried out by using GraphPad Prism version 9.4.1 for Windows (GraphPad Software, San Diego, California USA, www.graphpad.com).

#### 4.2.4 Determination of the Antibiofilm Activity

The ability of some selected compounds to inhibit biofilm formation was assessed by established protocols with minor modifications [34]. In short, exponential cultures of *S. aureus*, *E. coli*, and *C. albicans* grown in Tryptic Soy broth (TSB, Sigma‐Aldrich, St. Louis, MO, USA) were adjusted to a final density of 10^5^ CFU/mL. Aliquots of 100 µL of the microbial cultures were transferred to a 96‐well flat‐bottom polystyrene microplate, and then incubated at 37°C for 90 min to promote bacterial adhesion. Thereafter, wells were slowly rinsed with PBS to remove nonattached cells, and 100 µL of TSB supplemented with 1% glucose containing the twofold serial dilutions of the compounds was added. After incubation at 37°C for 24 h, the free‐floating cells were removed, and the biofilms were carefully washed with PBS. The biomass was quantified by a standardized crystal violet (CV) staining. Briefly, 100 µL of CV solution (0.1% in water) was added to each well containing a completely air‐dried biofilm, and incubated for 30 min at 37°C. Then, wells were washed twice with water to remove the unbound dye and CV was dissolved with 100 µL of 95% ethanol for 30 min. Finally, the colored supernatants were transferred to a new microplate, and the OD_550nm_ was measured. The percentage inhibition of biofilm formation in the different experimental conditions was calculated relative to the biofilm mass formed in the absence of compounds (positive control).

#### 4.2.5 In Vitro Cytotoxicity Studies

##### 4.2.5.1 Hemolytic Activity Assay

The hemolytic activity of some selected compounds was evaluated as the amount of hemoglobin released by the disruption of human red blood cells (hRBCs) [30]. For the experiments, fresh hRBCs were collected by centrifugation at 1500*g* for 10 min, washed three times with PBS, and resuspended to a final concentration of 4% w/v hRBCs in PBS. Then, 100 µL of hRBCs suspension and an equal volume of the twofold dilutions of the antimicrobial peptides were mixed in a 96‐well plate and incubated for 90 min at 37°C. After centrifugation at 1000*g* for 5 min, the supernatants were transferred into a clear 96‐well plate, and OD_405nm_ was read. Untreated hRBCs (incubated with PBS) and hRBCs incubated with 1% Triton X‐100 were employed as negative and positive controls, respectively. The hemolysis percentage was calculated as [OD_405nm_ (sample) − OD4_05nm_ (negative control)]/[OD_405nm_ (positive control) − OD_405nm_ (negative control)] × 100. Minimal hemolytic concentrations (MHCs) were defined as the compound concentration causing 10% hemolysis. Three independent experiments were performed in triplicate.

##### 4.2.5.2 Cell Viability and Proliferation Assay

Vero cell line (ATCC CCL‐81) was selected as a model system to investigate the overall effect of chalcones on non‐malignant mammalian cells. Briefly, cells were cultured in RPMI‐1640 medium supplemented with 10% fetal bovine serum (FBS) (Carlo Erba Reagents, Milan, Italy), 100 U/mL penicillin, and 100 µg/mL streptomycin at 37°C with 5% CO_2_. For experiments, cells were seeded into 96‐well plates at 10^4^ cells/well and incubated at 37°C for 24 h. Following washes with PBS, the cell monolayer was incubated with 100 µL of medium containing the twofold serial dilutions of the compounds. Both untreated cells and cells incubated with medium containing solvent dilutions were included in each experiment as controls. The cell viability was assessed by a WST8‐based assay according to the manufacturer's instructions (CCK‐8, Cell Counting Kit‐8, Dojindo Molecular Technologies, Rockville, MD, USA). After 48 h of incubation, culture medium was removed from each well, the monolayer was washed with PBS, and 100 µL of fresh medium containing 10 µL of CCK‐8 solution was added. Following a 2 h incubation at 37°C, the OD_450nm_ was read, and data were expressed as the percentage of cell viability relative to the untreated controls. The CC_50_ was obtained on the corresponding dose–response curves generated as previously reported for IC_50_ values. Experiments were carried out in triplicate in three biological replicates.

### 4.3 Modeling Experimental Section

#### 4.3.1 Setup of MD Simulations

All‐atom MD simulations were performed using GROMACS 23 [35, 36]. QPySs **2** and **5** were modeled using the Charmm General Force Field (CGenFF) [37]. The simulations were carried out with a total of one molecule per system. Molecules were placed in the membrane through the Membrane Builder protocol from CHARMM‐GUI [38]. Gram‐positive membranes were modeled using a 1:3 ratio of 1‐palmitoyl‐2‐oleoyl‐phospatidylethanolamine (POPE) and 1‐Palmitoyl‐2‐oleoyl‐sn‐glycero‐3‐(phospho‐rac‐(1‐glycerol)) (POPG). Gram‐negative was modeled using the same lipid species in the 3:1 ratio. The systems were energy minimized and equilibrated for 10 and 50 ns for lipid membranes by following standard CHARMM‐GUI protocols and using CHARMM36 as the Force Field. In all cases, equilibration was achieved by monitoring the membrane area over the simulation. The periodic boundary conditions were used in all three directions. Atomic velocities and positions were updated with a time step of 2 fs employing the leapfrog integrator. All simulations were performed at 303.15 K using a Nosé‐Hoover thermostat [39] with a coupling constant of 1 ps. Semi‐isotropic pressure coupling was employed using the Parrinello‐Rahman barostat [40] with a coupling constant of 5 ps. The particle‐mesh Ewald (PME) [41] was chosen to calculate long‐range electrostatic interactions with a cutoff of 1.2 nm, while the linear constraint solver (LINCS) [42] was used for constraining hydrogen bonds. Verlet cutoff scheme is chosen to compute the nonbonded interactions (Lennard‐Jones 6‐12), which are truncated at the cutoff radius of 1.2 nm and shifted with a force‐switch function between 1.0 and 1.2 nm.

Each system was simulated for a total simulation time of 500 ns.

#### 4.3.2 Simulation of Diffusion of the Small Molecules

A pool of 50 independent simulations was performed for molecules **2** and **5** in both membrane models. For each simulation, the small molecules were placed 35 Å far from the membrane leaflet, approaching the lower boundary of the simulation box, as a starting configuration. Each simulation was run until the molecule's polar head center of mass (COM) approached the hydrophilic edge of the membrane. All the diffusion times were collected for statistical analysis. The Plumed software was used to control the simulations [43, 44, 45].

#### 4.3.3 Analysis and Visualisation

Visual molecular dynamics 1.9.3 (VMD) [46] is used for rendering MD snapshots. Analysis of QPySs conformations was computed using GROMACS post‐processing tools. Cluster analysis of dihedral angles was performed using the Gromos method [32], imposing an RMSD cut‐off of 1.25 Å. The three main clusters were also projected in a reduced space, gained from the principal component analysis (PCA) [47], performed on the QPySs polar head dihedral angles.

## Conflicts of Interest

The authors declare no conflicts of interest.

## Supporting information

Alkyl tail Var InChI: Novel compounds and Biological Screening Results.

Supplementary Alkyl Tail Variation‐REV: Dose‐response curves of the chalcone‐based QPySs (**2‐7**); Antibiofilm activity of the chalcone‐based QPySs (**4‐6**); Cluster Analysis of QPySs **2** and **5;** ^1^H and ^13^C NMR spectra of compounds **2**, **5**, and **11** can be found online.

## Data Availability

Data that support the findings of this study are openly available in Zenodo at https://zenodo.org/, reference number 10.5281/zenodo.12799976.
