# Supplementary material for: Alkyl Tail Variation on Chalcone‐Based Quaternary Pyridinium Salts as Rule‐of‐Thumb for Antimicrobial Activity
Source: Arch Pharm (Weinheim). 2025 May 11;358(5):e70003. doi: 10.1002/ardp.70003 (PMC12066977; doi:10.1002/ardp.70003)
Supplement: Supplementary file 1 — Alkyl tail Var InChI: Novel compounds and Biological Screening Results. [file ARDP-358-e70003-s001.docx]

**Supplemental Material: Novel Compounds and Biological Screening Results**

**Title of Manuscript**

Alkyl Tail Variation on Chalcone-based Quaternary Pyridinium Salts as Rule-of-Thumb for Antimicrobial Activity

**Authors**

Francesca Seghetti^1§^^, Riccardo Ocello^1,2^^, Alessandra Bisi^1^, Matteo Masetti^1^, Silvia Gobbi^1^, Federico Falchi^1,2^, Giovanna Angela Gentilomi^3,4^, Francesca Bonvicini^3*^, Federica Belluti^1*^

**Affiliations**

^1^Department of Pharmacy and Biotechnology, *Alma Mater Studiorum*-University of Bologna, Via Belmeloro, 6, 40126 Bologna, Italy.

^2^Computational and Chemical Biology, Italian Institute of Technology IIT, via Morego 30, 16163 Genoa, Italy.

^3^Department of Pharmacy and Biotechnology, *Alma Mater Studiorum*-University of Bologna, Via Massarenti 9, 40138 Bologna, Italy.

^4^Microbiology Unit, IRCCS Azienda Ospedaliero-Universitaria di Bologna, Via Massarenti 9, Bologna, 40138, Italy.

Present address: ^§^ Aptuit, an Evotec Company

**Corresponding authors**

Federica Belluti, Department of Pharmacy and Biotechnology, *Alma Mater Studiorum*-University of Bologna, Via Belmeloro, 6, 40126 Bologna, Italy. Email: [federica.belluti@unibo.it](mailto:federica.belluti@unibo.it) Tel: +39 051 2099701.

Francesca Bonvicini, Department of Pharmacy and Biotechnology, *Alma Mater Studiorum*-University of Bologna, Via Massarenti 9, 40138 Bologna, Italy. Email: [francesca.bonvicini4@unibo.it](mailto:francesca.bonvicini4@unibo.it), Tel: +39 051 4290930.

| **Compound No.** | **InChI** | **Biological Activity (MIC values for *S. aureus*, *E. coli*, and *C. albicans*)^a^** |
| --- | --- | --- |
| **1** | InChI=1S/C20H15NO3/c1-3-12-23-17-8-9-18(20(14-17)24-13-4-2)19(22)10-7-16-6-5-11-21-15-16/h1-2,5-11,14-15H,12-13H2/b10-7+ | *S. aureus* 100 µM  *E. coli >*100 µM  *C. albicans* 100 µM |
| **2** | InChI=1S/C26H28NO3.BrH/c1-4-7-8-9-16-27-17-10-11-22(21-27)12-15-25(28)24-14-13-23(29-18-5-2)20-26(24)30-19-6-3;/h2-3,10-15,17,20-21H,4,7-9,16,18-19H2,1H3;1H/q+1;/p-1/b15-12+; | *S. aureus* 50 µM  *E. coli >*100 µM  *C. albicans* 100 µM |
| **3** | InChI=1S/C28H32NO3.BrH/c1-4-7-8-9-10-11-18-29-19-12-13-24(23-29)14-17-27(30)26-16-15-25(31-20-5-2)22-28(26)32-21-6-3;/h2-3,12-17,19,22-23H,4,7-11,18,20-21H2,1H3;1H/q+1;/p-1/b17-14+; | *S. aureus* 6.25 µM  *E. coli* 12.5 µ*M*  *C. albicans* 50 µM |
| **4** | InChI=1S/C30H36NO3.BrH/c1-4-7-8-9-10-11-12-13-20-31-21-14-15-26(25-31)16-19-29(32)28-18-17-27(33-22-5-2)24-30(28)34-23-6-3;/h2-3,14-19,21,24-25H,4,7-13,20,22-23H2,1H3;1H/q+1;/p-1/b19-16+; | *S. aureus* 1.56 µM  *E. coli* 6.25 µM  *C. albicans* 6.25 µM |
| **5** | InChI=1S/C32H40NO3.BrH/c1-4-7-8-9-10-11-12-13-14-15-22-33-23-16-17-28(27-33)18-21-31(34)30-20-19-29(35-24-5-2)26-32(30)36-25-6-3;/h2-3,16-21,23,26-27H,4,7-15,22,24-25H2,1H3;1H/q+1;/p-1/b21-18+; | *S. aureus* 3.125 µM  *E. coli* 6.25 µM  *C. albicans* 3.125 µM |
| **6** | InChI=1S/C34H44NO3.BrH/c1-4-7-8-9-10-11-12-13-14-15-16-17-24-35-25-18-19-30(29-35)20-23-33(36)32-22-21-31(37-26-5-2)28-34(32)38-27-6-3;/h2-3,18-23,25,28-29H,4,7-17,24,26-27H2,1H3;1H/q+1;/p-1/b23-20+; | *S. aureus* 3.125 µM  *E. coli* 50 µM  *C. albicans* 1.56 µM |
| **7** | InChI=1S/C36H48NO3.BrH/c1-4-7-8-9-10-11-12-13-14-15-16-17-18-19-26-37-27-20-21-32(31-37)22-25-35(38)34-24-23-33(39-28-5-2)30-36(34)40-29-6-3;/h2-3,20-25,27,30-31H,4,7-19,26,28-29H2,1H3;1H/q+1;/p-1/b25-22+; | *S. aureus* 12.5 µM  *E. coli >*100 µM  *C. albicans* 6.25 µM |
| **8** | InChI=1S/C38H52NO3.BrH/c1-4-7-8-9-10-11-12-13-14-15-16-17-18-19-20-21-28-39-29-22-23-34(33-39)24-27-37(40)36-26-25-35(41-30-5-2)32-38(36)42-31-6-3;/h2-3,22-27,29,32-33H,4,7-21,28,30-31H2,1H3;1H/q+1;/p-1/b27-24+; | *S. aureus* 100 µM  *E. coli >*100 µM  *C. albicans* *>*100 µM |
| **9** | InChI=1S/C20H15NO3.Br/c1-3-12-23-17-8-9-18(20(14-17)24-13-4-2)19(22)10-7-16-6-5-11-21-15-16;/h1-2,5-10,14-15,21H,12-13H2;/b10-7+; | *S. aureus >*100 µM  *E. coli >*100 µM  *C. albicans* >100 µM |
| **10** | InChI=1S/C46H42N2O6/c1-5-29-51-39-19-21-41(45(33-39)53-31-7-3)43(49)23-17-37-15-13-27-47(35-37)25-11-9-10-12-26-48-28-14-16-38(36-48)18-24-44(50)42-22-20-40(52-30-6-2)34-46(42)54-32-8-4/h1-4,13-24,27-28,33-36H,9-12,25-26,29-32H2/q+2/b23-17+,24-18+ | *S. aureus* 50 µM  *E. coli* 50 µM  *C. albicans* 100 µM |
| **11** | InChI=1S/C22H40N.BrH/c1-3-4-5-6-7-8-9-10-11-12-13-14-15-16-19-23-20-17-18-22(2)21-23;/h17-18,20-21H,3-16,19H2,1-2H3;1H/q+1;/p-1 | *S. aureus* 6.25 µM  *E. coli* 12.5 µM  *C. albicans* 6.25 µM |

^a^ MIC (Minimum Inhibitory Concentration) were determined *in vitro* by means of a standardized microdilution assay, in compliance with the Clinical and laboratory Standard Institute (CLSI) guidelines (30). Microbial suspensions were prepared at 0.5 McFarland in PBS, then diluted in the media and transferred in a 96 micro-well plate together with the serially 2-fold dilutions of the compounds. After 24h of incubation at 37°C, microbial growths were evaluated by measuring the Optical Density at 600 nm, then percentage values were obtained as relative to the positive controls (microbial suspensions in regular media). MIC was defined as the lowest concentration of compounds that inhibits the growth of microorganisms (<10%).
