# Supplementary material for: Alkyl Tail Variation on Chalcone‐Based Quaternary Pyridinium Salts as Rule‐of‐Thumb for Antimicrobial Activity
Source: Arch Pharm (Weinheim). 2025 May 11;358(5):e70003. doi: 10.1002/ardp.70003 (PMC12066977; doi:10.1002/ardp.70003)
Supplement: Supplementary file 2 — Supplementary Alkyl Tail Variation‐REV: Dose‐response curves of the chalcone‐based QPySs (2‐7); Antibiofilm activity of the chalcone‐based QPySs (4‐6); Cluster Analysis of QPySs 2 and 5; 1H and 13C NMR spectra of compounds 2, 5, and 11 can be found online. [file ARDP-358-e70003-s002.pdf]

**Alkyl Tail Variation on Chalcone-based Quaternary Pyridinium Salts as Rule-of-Thumb for Antimicrobial Activity**

Francesca Seghetti[1§^], Riccardo Ocello[1,2^], Alessandra Bisi[1], Matteo Masetti[1], Silvia Gobbi[1], Federico Falchi[1,2], Giovanna Angela Gentilomi[3,4], Francesca Bonvicini[3*], Federica Belluti[1*]

[1]Department of Pharmacy and Biotechnology, *Alma Mater Studiorum*-University of Bologna, Via Belmeloro, 6, 40126 Bologna, Italy.
[2]Computational and Chemical Biology, Italian Institute of Technology IIT, via Morego 30, 16163 Genoa, Italy.
[3]Department of Pharmacy and Biotechnology, *Alma Mater Studiorum*-University of Bologna, Via Massarenti 9, 40138 Bologna, Italy.
[4]Microbiology Unit, IRCCS Azienda Ospedaliero-Universitaria di Bologna, Via Massarenti 9, Bologna, 40138, Italy.

Present address: [§] Aptuit, an Evotec Company, Via Alessandro Fleming, 4, Verona 37135, Italy.

^These authors contributed equally to this work
*Corresponding authors: francesca.bonvicini4@unibo.it, Tel: +39 051 4290930; federica.belluti@unibo.it Tel: +39 051 2099701.

**Contents**

**Figure S1.** Dose-response curves of the active chalcone-based QPySs on the tested reference strains. .................. S1
**Figure S2.** Antibiofilm activity of the chalcone-based QPySs (**4-6**) on the tested reference strains. .................. S2
**Figure S3.** Cluster Analysis of QPySs **2** and **5**. .................. S3
**Figure S4.** $^{1}$H NMR of compound **1** **S5**
**Figure S5.** $^{13}$C NMR of compound **1** **S5**
**Figure S6.** $^{1}$H NMR of compound **2** **S6**
**Figure S7.** $^{13}$C NMR of compound **2** **S6**
**Figure S8.** $^{1}$H NMR of compound **3** **S7**
**Figure S9.** $^{13}$C NMR of compound **3** **S7**
**Figure S10.** $^{1}$H NMR of compound **4** **S8**
**Figure S11.** $^{13}$C NMR of compound **4** **S8**
**Figure S12.** $^{1}$H NMR of compound **5** **S9**
**Figure S13.** $^{13}$C NMR of compound **5** **S9**
**Figure S14.** $^{1}$H NMR of compound **6** **S10**
**Figure S15.** $^{13}$C NMR of compound **6** **S10**
**Figure S16.** $^{1}$H NMR of compound **7** **S11**
**Figure S17.** $^{13}$C NMR of compound **7** **S12**
**Figure S18.** $^{1}$H NMR of compound **8** **S13**
**Figure S19.** $^{13}$C NMR of compound **8** **S13**
**Figure S20.** $^{1}$H NMR of compound **9** **S14**
**Figure S21.** $^{13}$C NMR of compound **9** **S14**
**Figure S22.** $^{1}$H NMR of compound **10** **S15**
**Figure S23.** $^{13}$C NMR of compound **10** **S15**
**Figure S24.** $^{1}$H NMR of compound **11** **S16**
**Figure S25.** $^{13}$C NMR of compound **11** **S16**

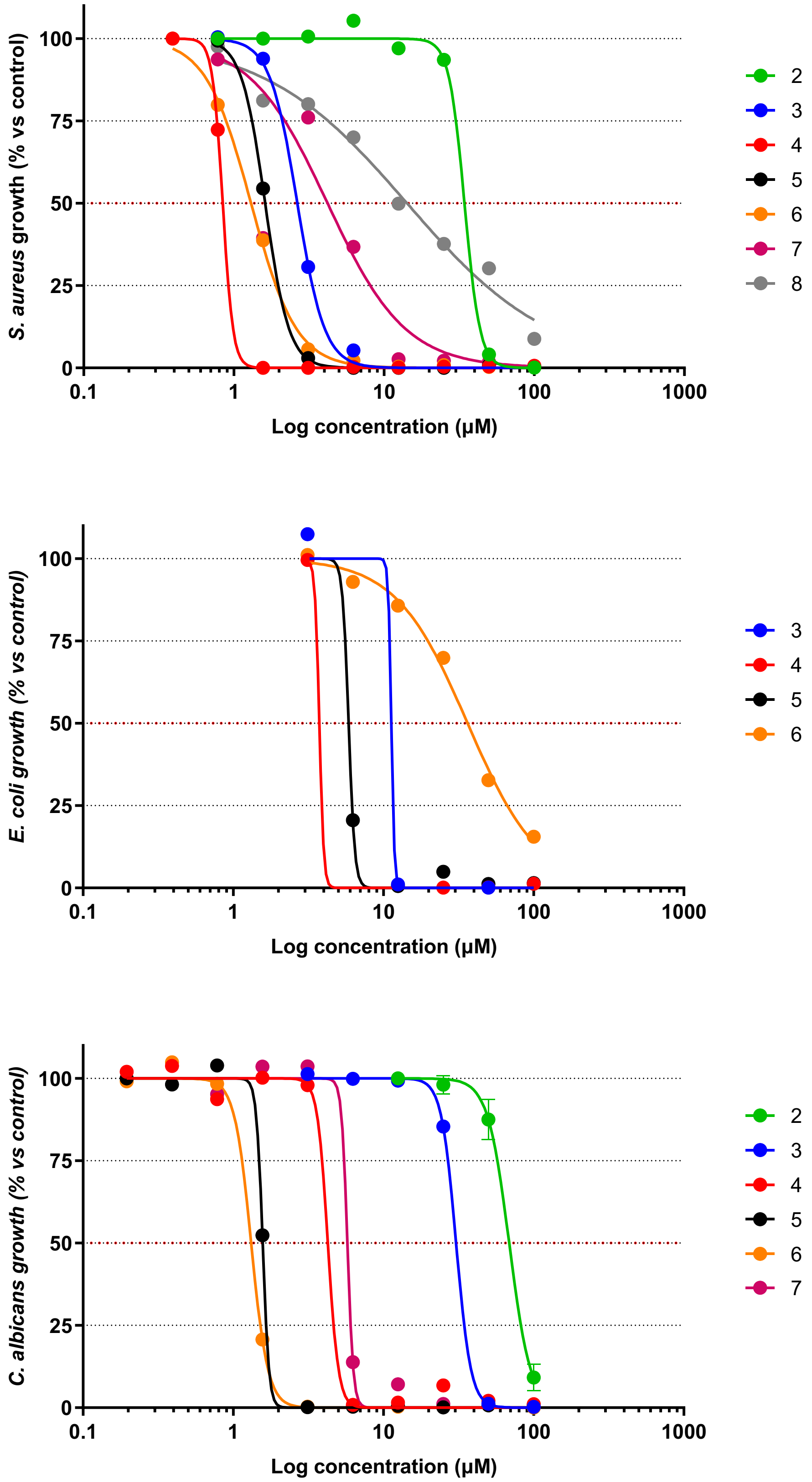


**Figure S1. Dose-response curves of the active chalcone-based QPySs on the tested reference strains**. Curves are obtained by using a nonlinear regression equation and a variable slope model (log(inhibitor) vs. normalised response -- Variable slope (GraphPad Prism version 9.4.1 for Windows).

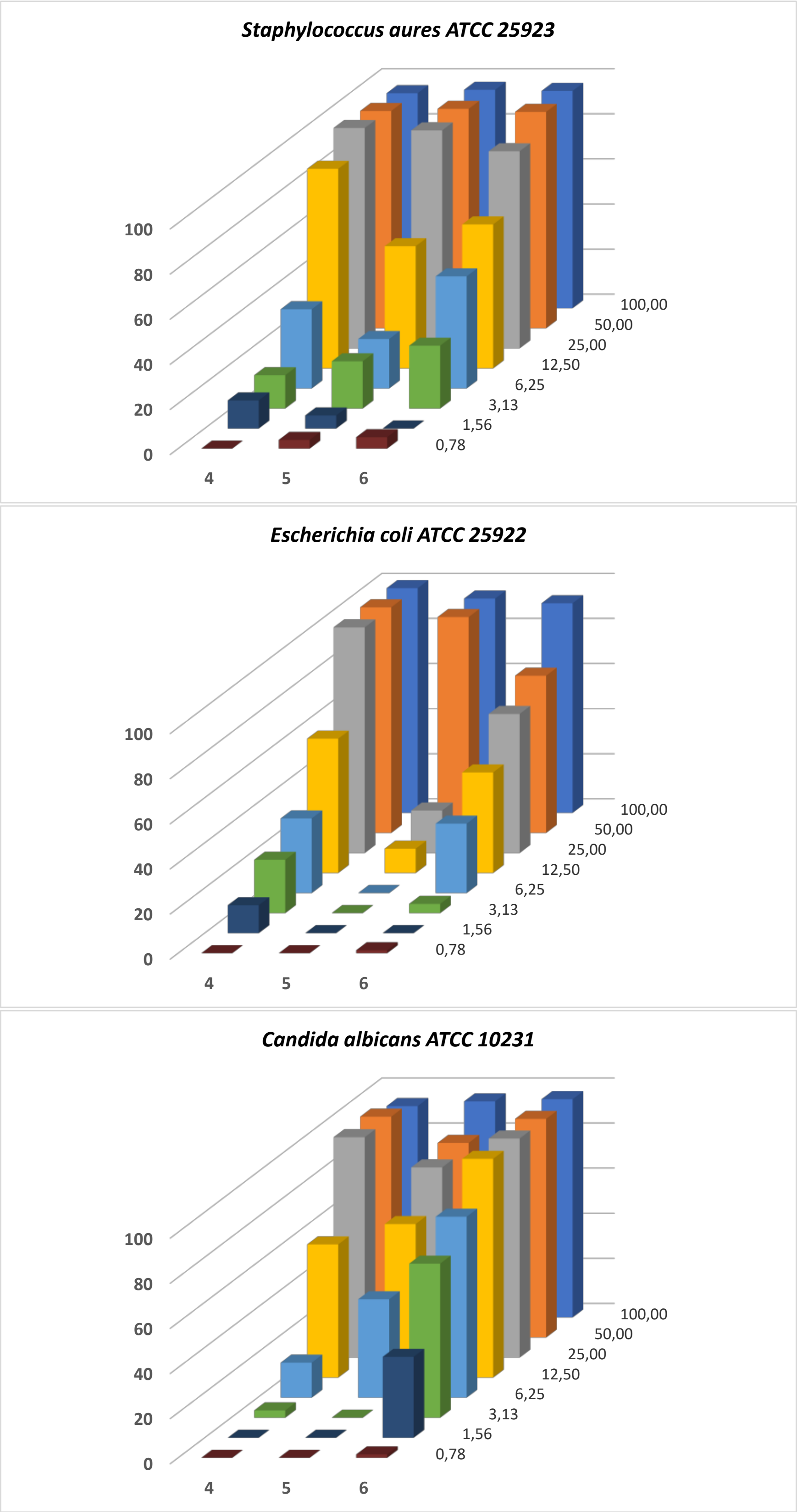


**Figure S2. Antibiofilm activity of the chalcone-based QPySs (4-6) on the tested reference strains.** Data are the mean percentage values of the biofilm biomass determined by CV staining of the compounds at the different concentrations relative to the untreated biofilms.

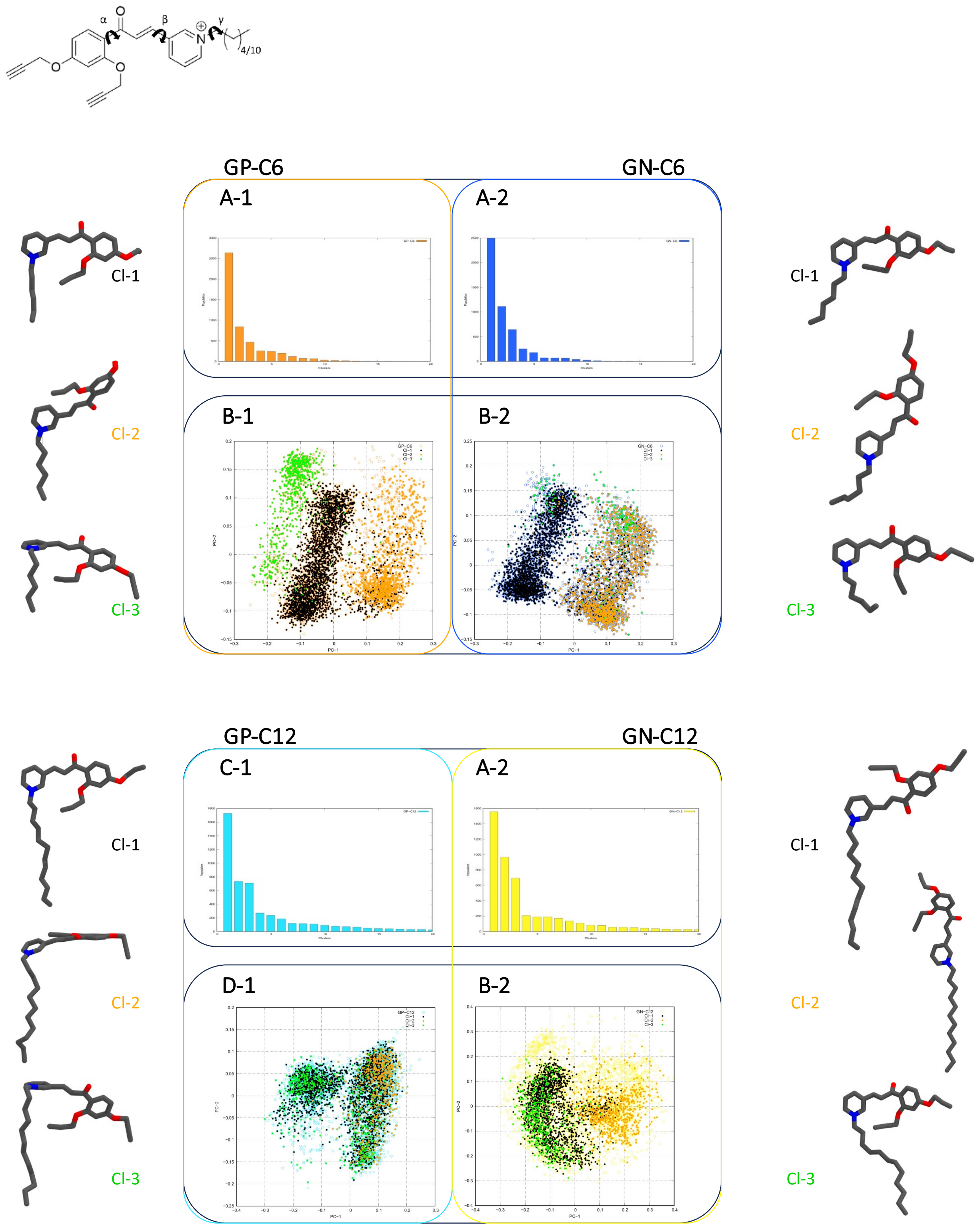


**Figure S3. Cluster Analysis of QPySs 2 and 5**. All dihedral angles were considered to cluster the ligands. The Gromos method was used adopting a 1.25 Å for the RMSD cut-off. QPySs structures corresponding to the three main clusters are represented for comparison. Each structure belonging to the three main clusters is also projected on the first two components obtained from a Principal Component Analysis performed considering α,β,γ dihedral angles.

**NMR spectra**

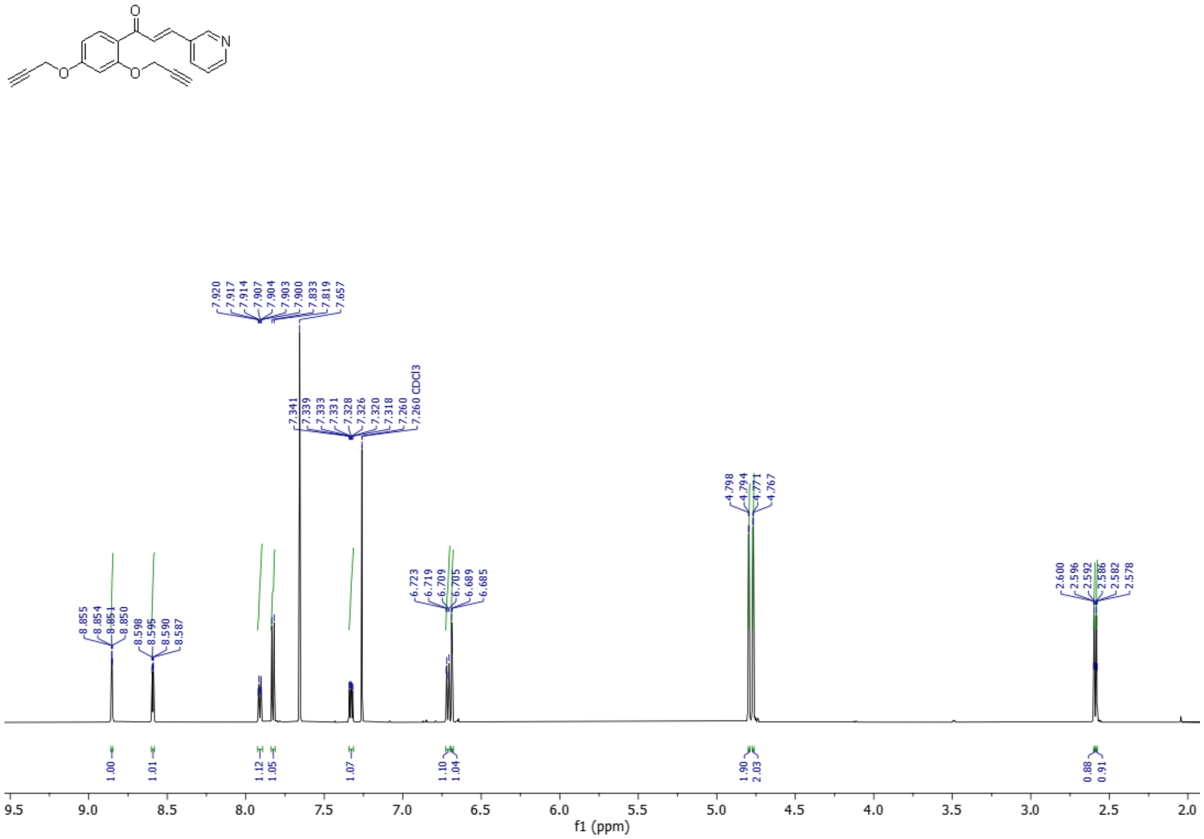


**Figure S4. $^{1}$H NMR spectrum** (600 MHz, $CDCl_3$) of compound **1**.

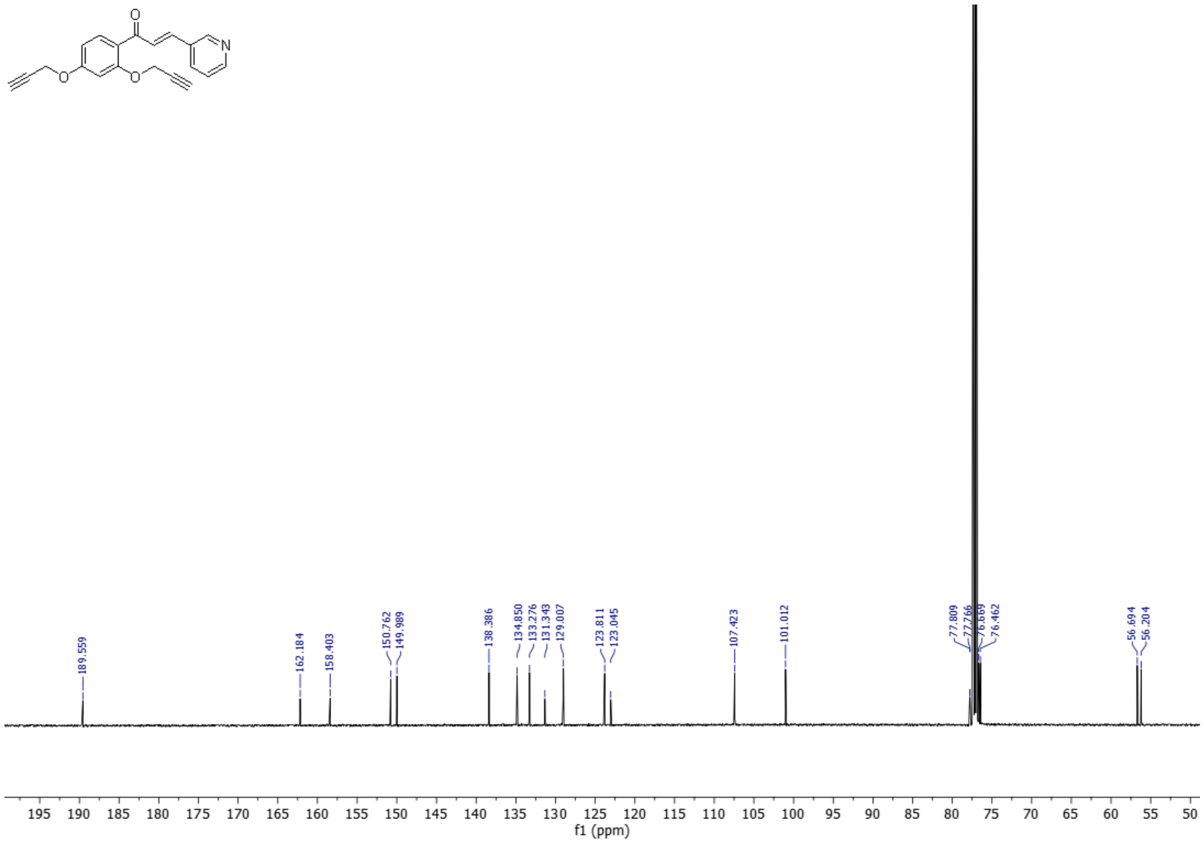


**Figure S5. $^{13}$C NMR spectrum** (150 MHz, $CDCl_3$) of compound **1**.

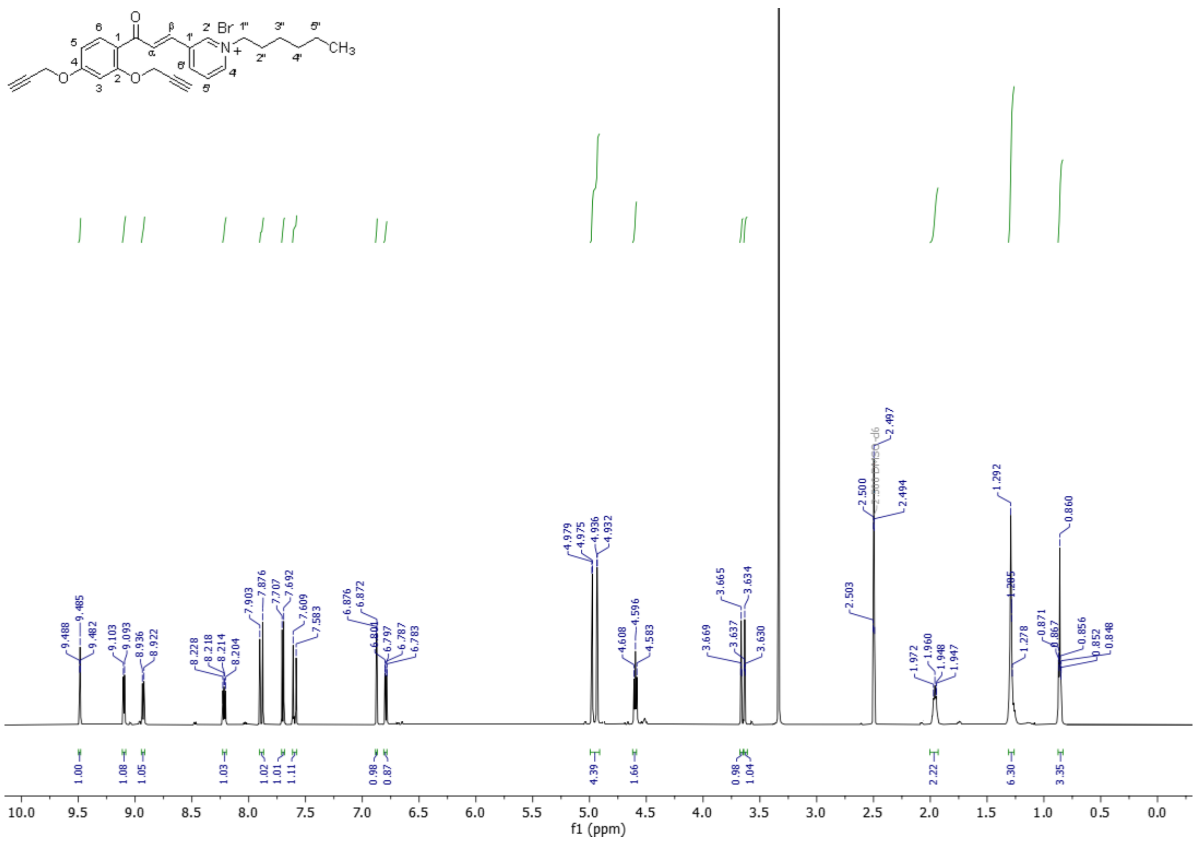

**Figure S6.** **¹H NMR spectrum** (600 MHz, DMSO-d6) of compound **2**.

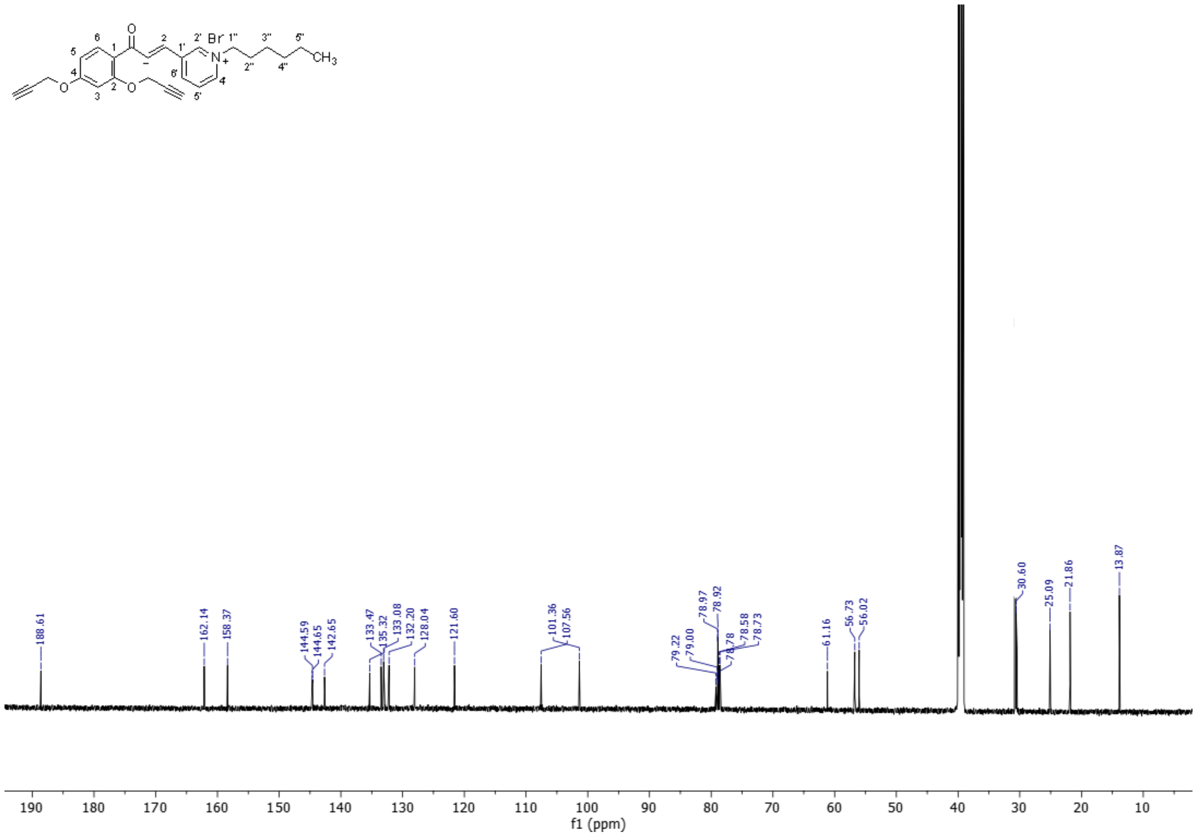

**Figure S7.** **¹³C NMR spectrum** (151 MHz, DMSO-d6) of compound **2**.

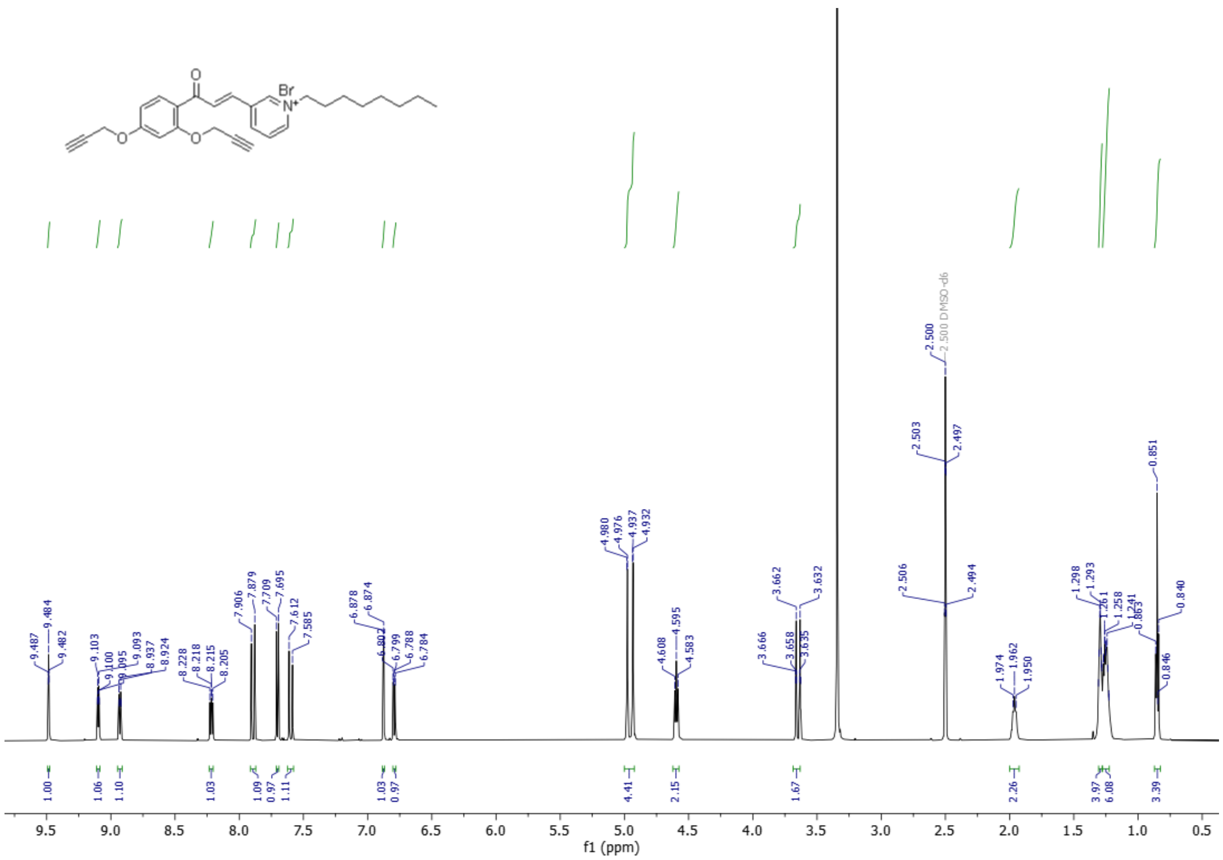

**Figure S8. $^{1}$H NMR spectrum** (600 MHz, DMSO-d$_6$) of compound **3**.

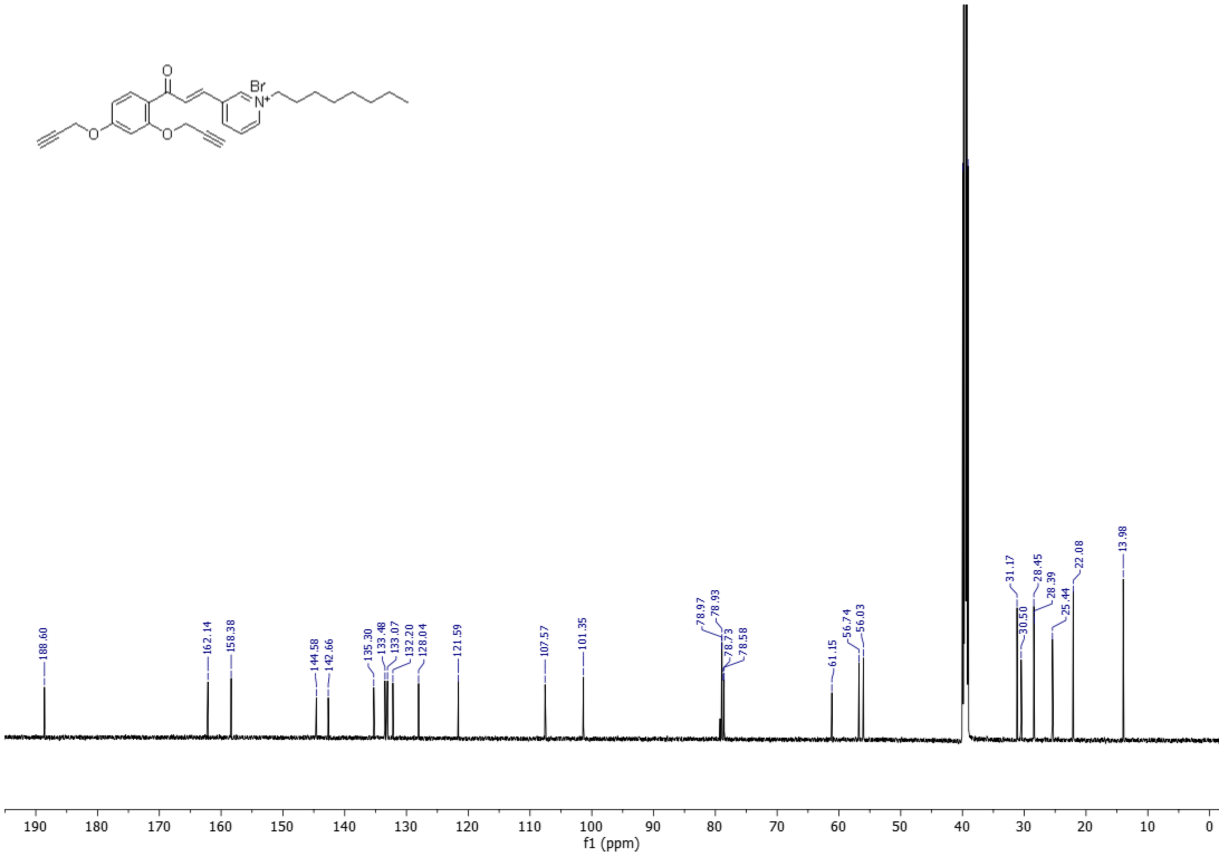

**Figure S9. $^{13}$C NMR spectr*um*** (151 MHz, DMSO-d$_6$) of compound **3**.

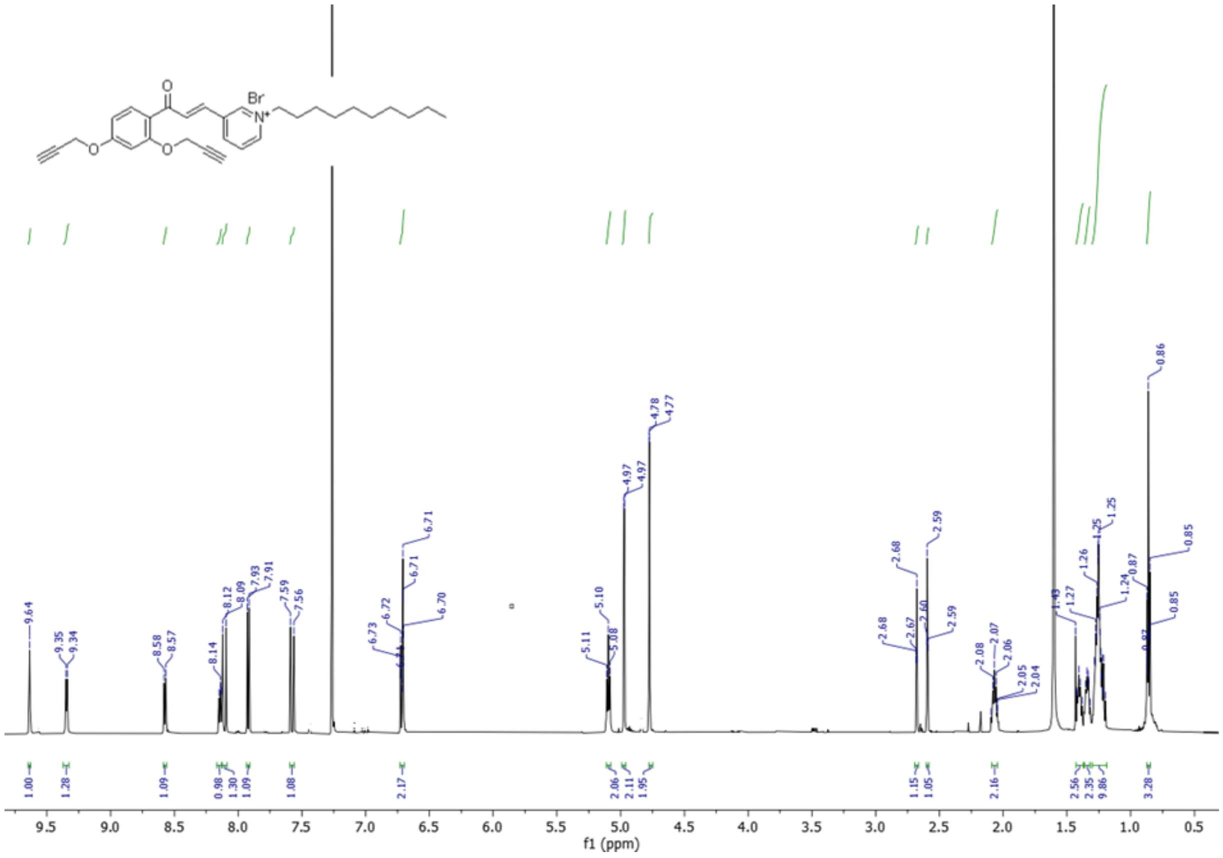

**Figure S10.** **$^1$H NMR spectrum** (600 MHz, $CDCl_3$) of compound **4**

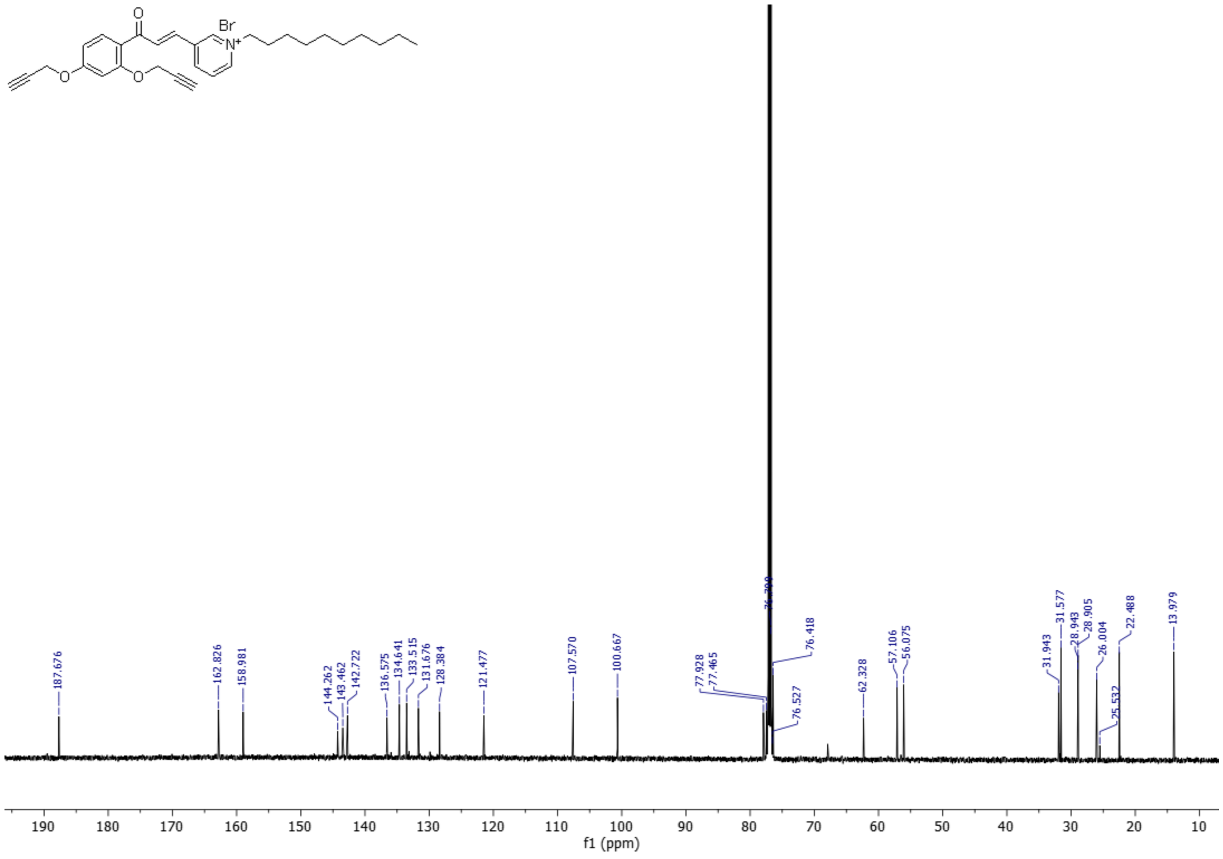

**Figure S11.** **$^{13}$C NMR spectrum** (151 MHz, $CDCl_3$) of compound **4**

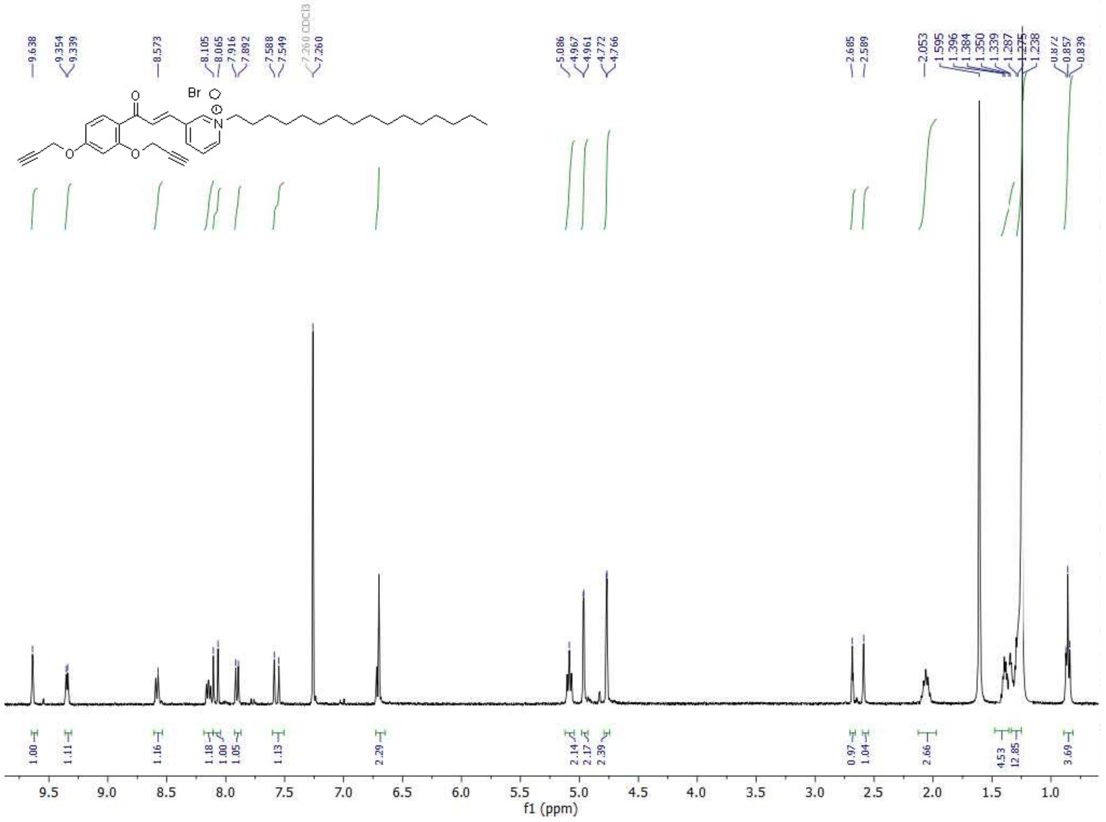


**Figure S12. $^{1}$H NMR spectrum** (400 MHz, $CDCl_3$) of compound **5**.

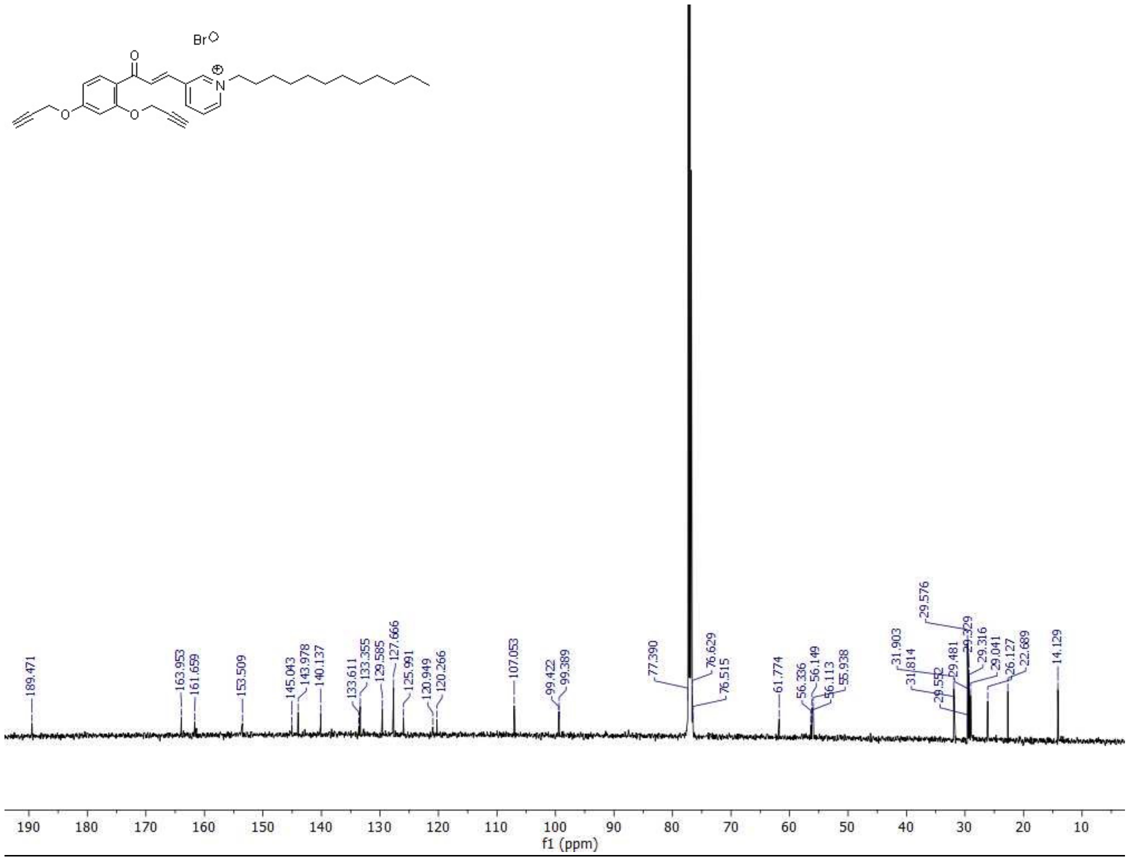


**Figure S13. $^{13}$C NMR spectrum** (400 MHz, $CDCl_3$) of compound **5**.

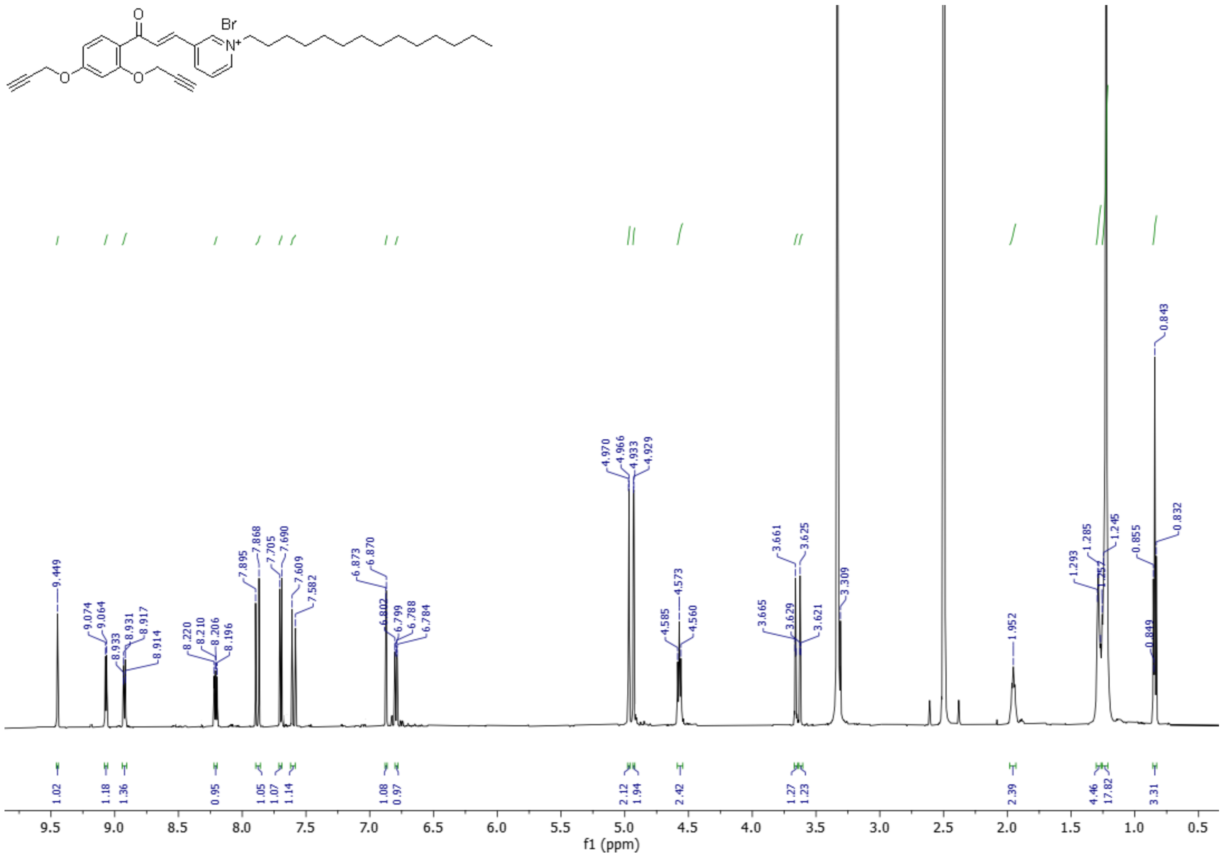


**Figure S14.** **$^{1}$H NMR spectr*um*** (600 MHz, $CDCl_3$) of compound **6.**

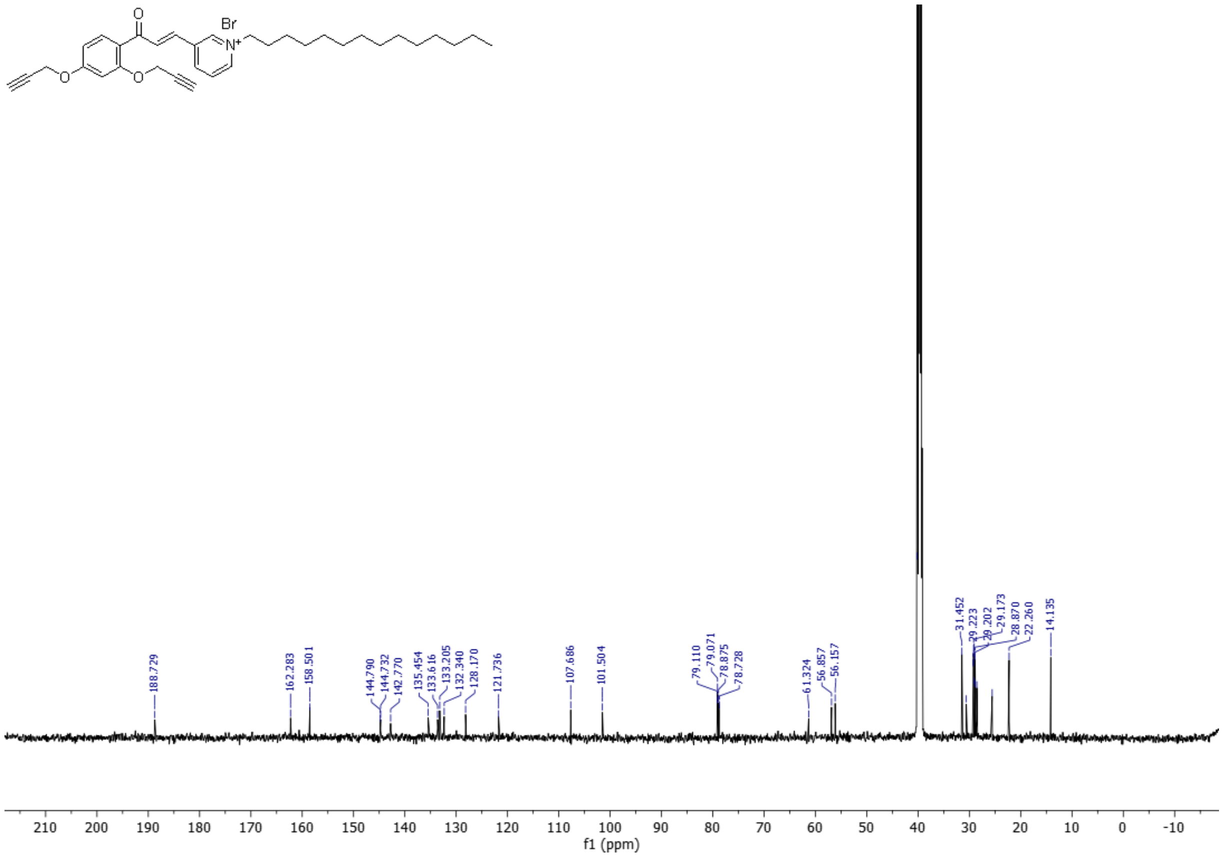


**Figure S15.** **$^{13}$C NMR spectr*um*** (151 MHz, $CDCl_3$) of compound **6.**

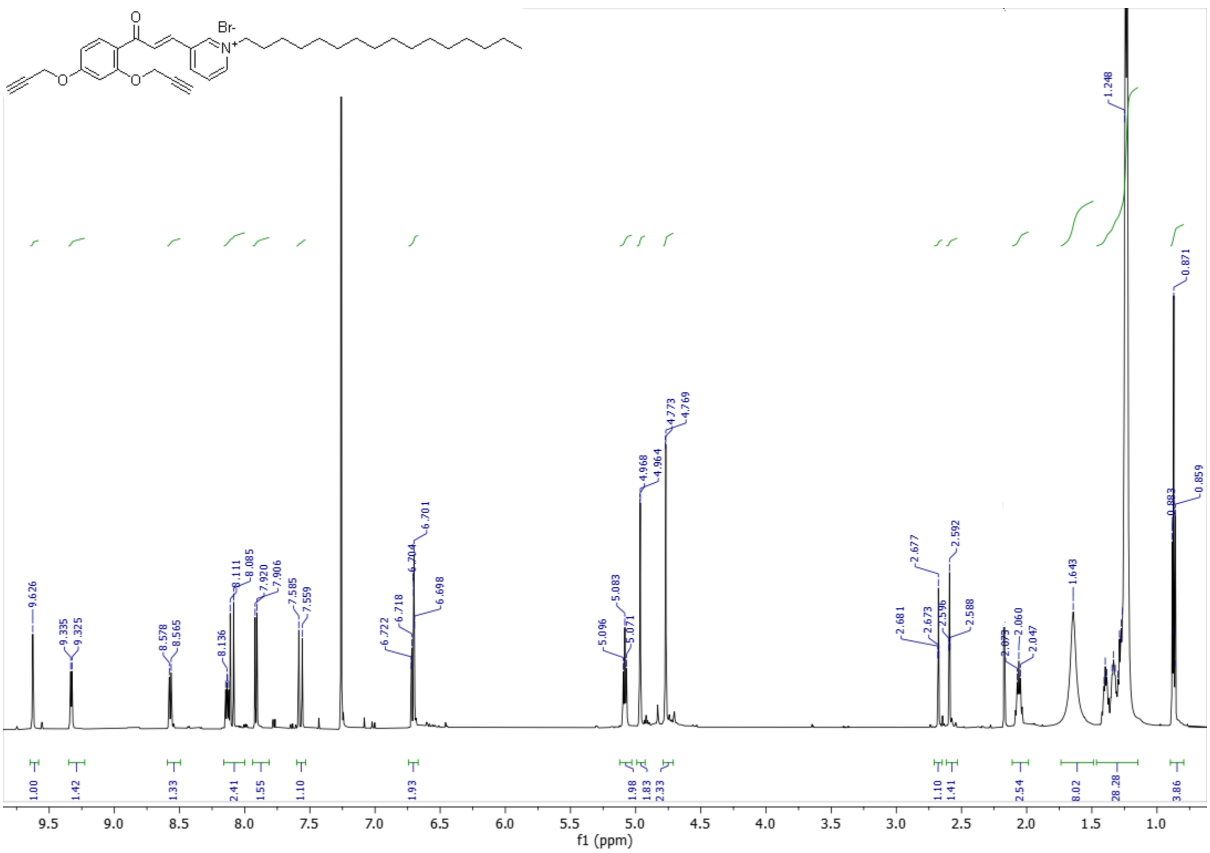

**Figure S16.** **$^1$H NMR spectr*um*** (600 MHz, $CDCl_3$) of compound **7.**

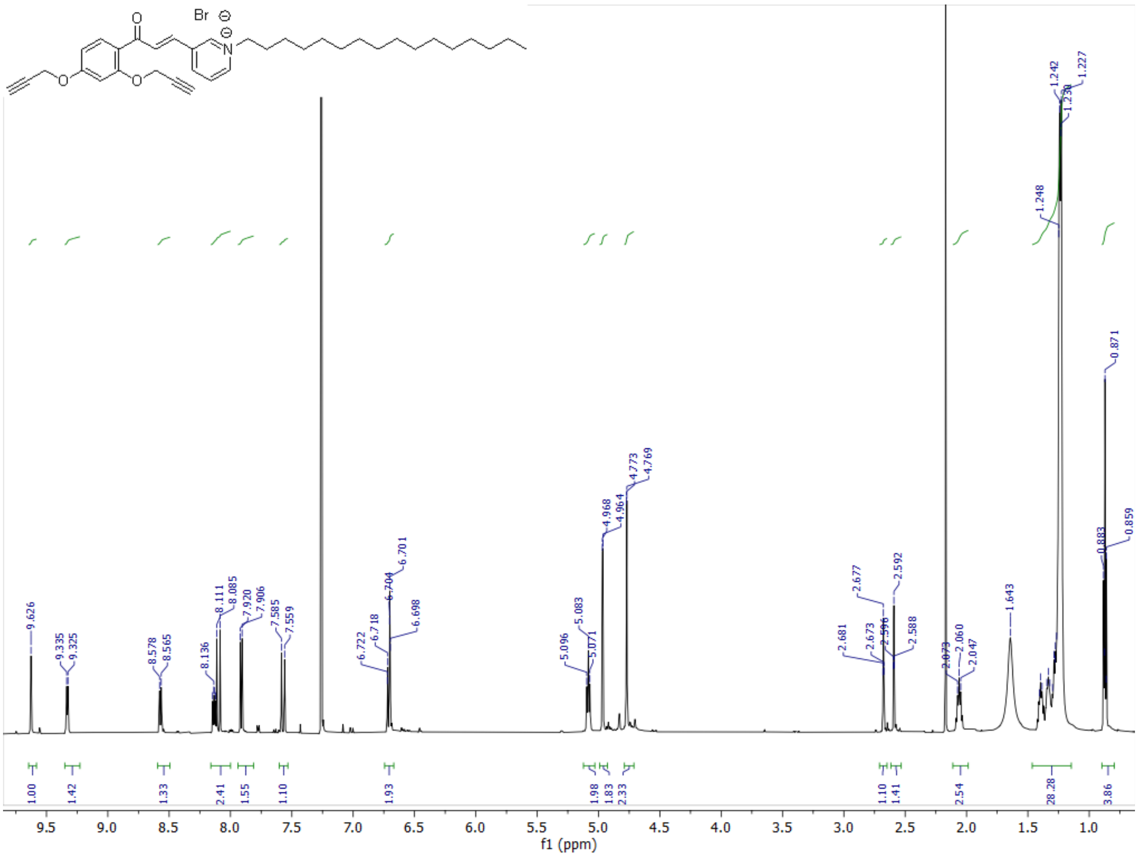

**Figure S17.** **$^{13}$C NMR spectr*um*** (151 MHz, $CDCl_3$) of compound **7**

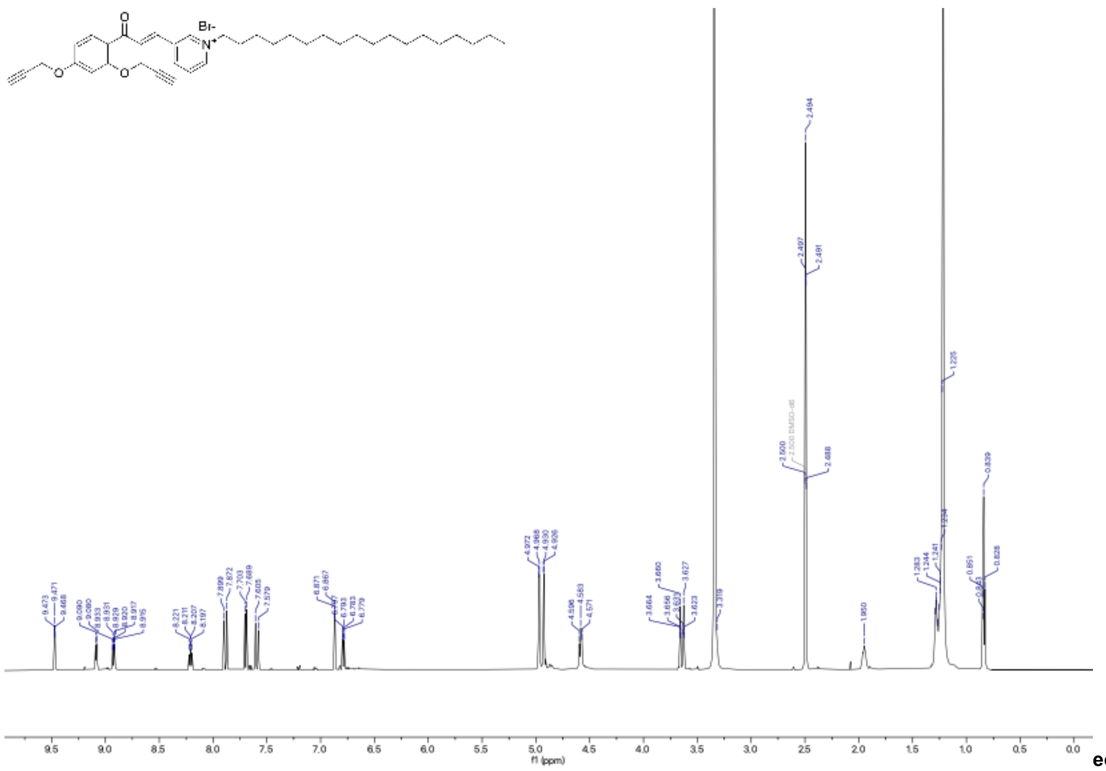


ectra (600 MHz,

**Figure S18.** **¹H NMR spectrum** (600 MHz, DMSO-d$_6$) of compound **8**

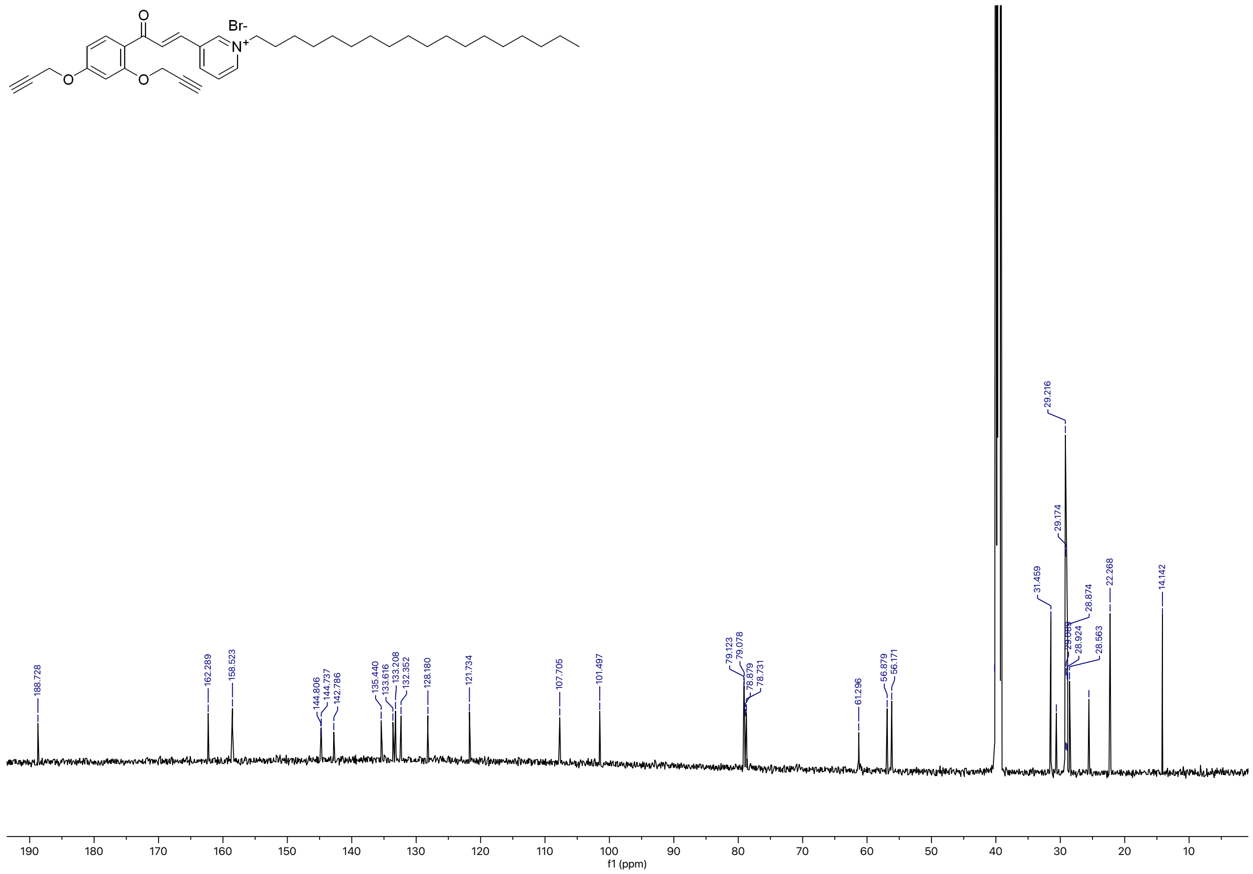


**Figure S19.** **¹³C NMR spectr*um*** (151 MHz, DMSO-d$_6$) of compound **8**

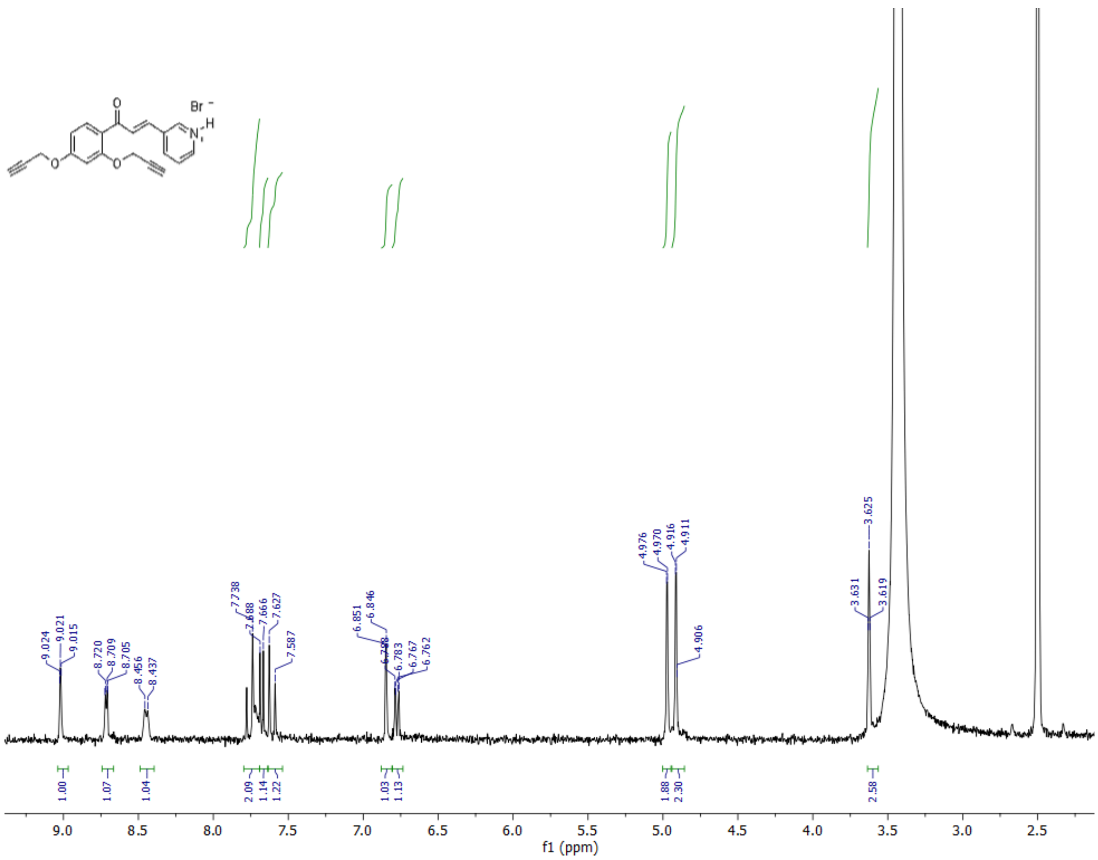

**Figure S20.** **$^{1}$H NMR spectr*um*** (400 MHz, DMSO-$d_6$) of compound **9**

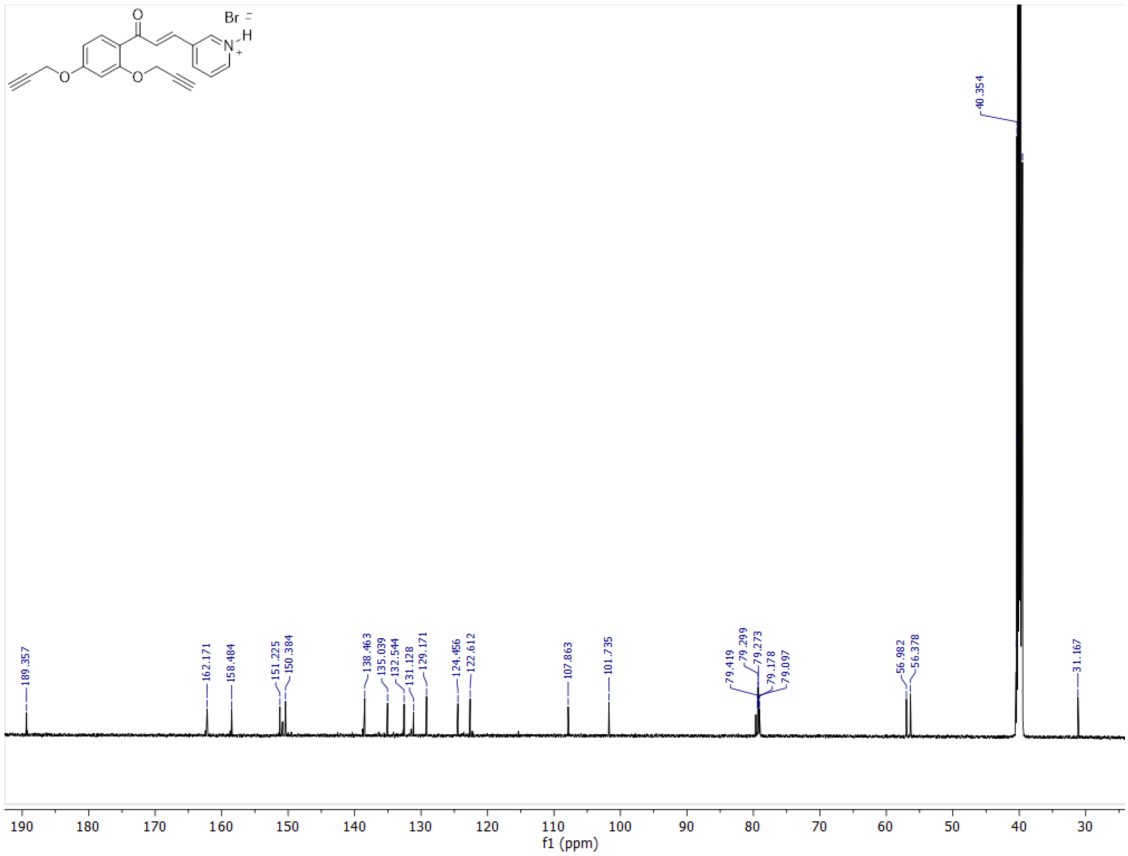

**Figure S21.** **$^{13}$C NMR spectr*um*** (151 MHz, $CDCl_3$) of compound **9**

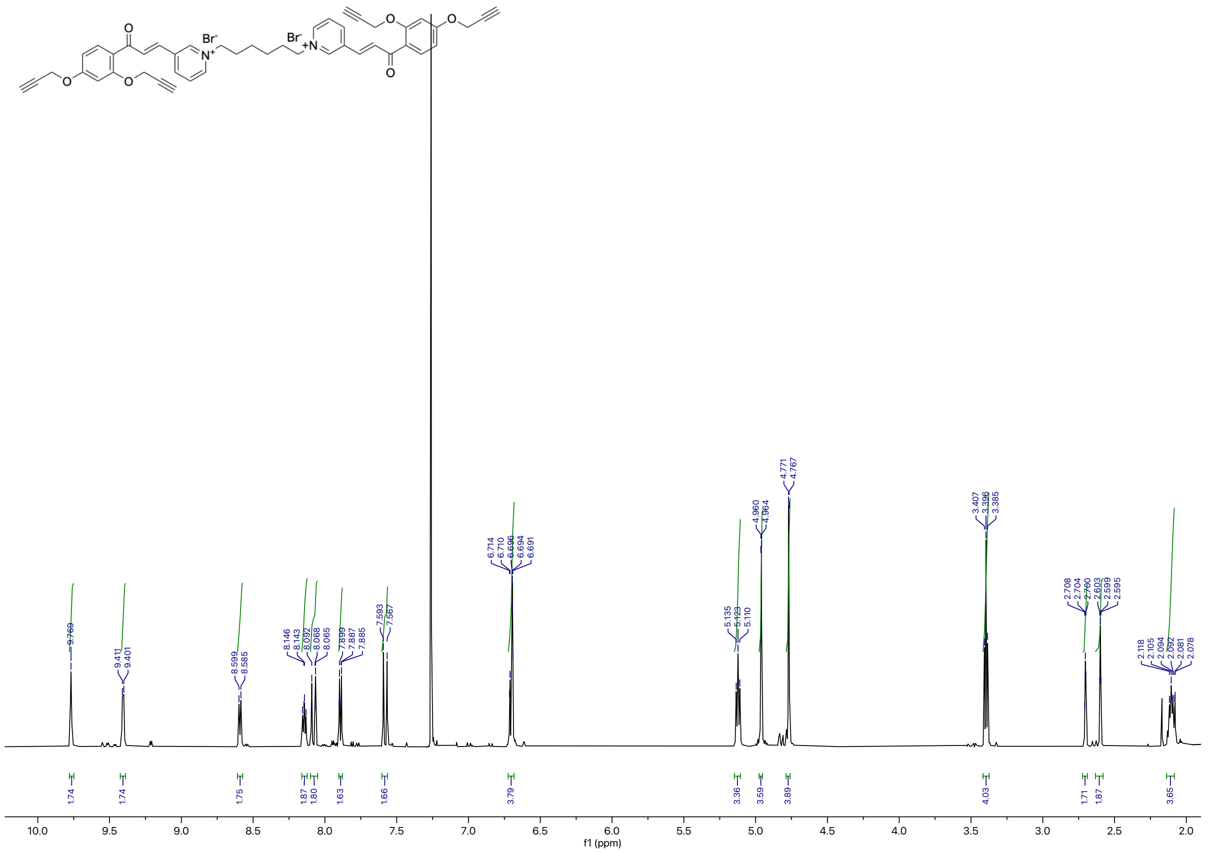


**Figure S22.** $^{1}$**H NMR spectr*um*** (600 MHz, $CDCl_3$) of compound **10.**

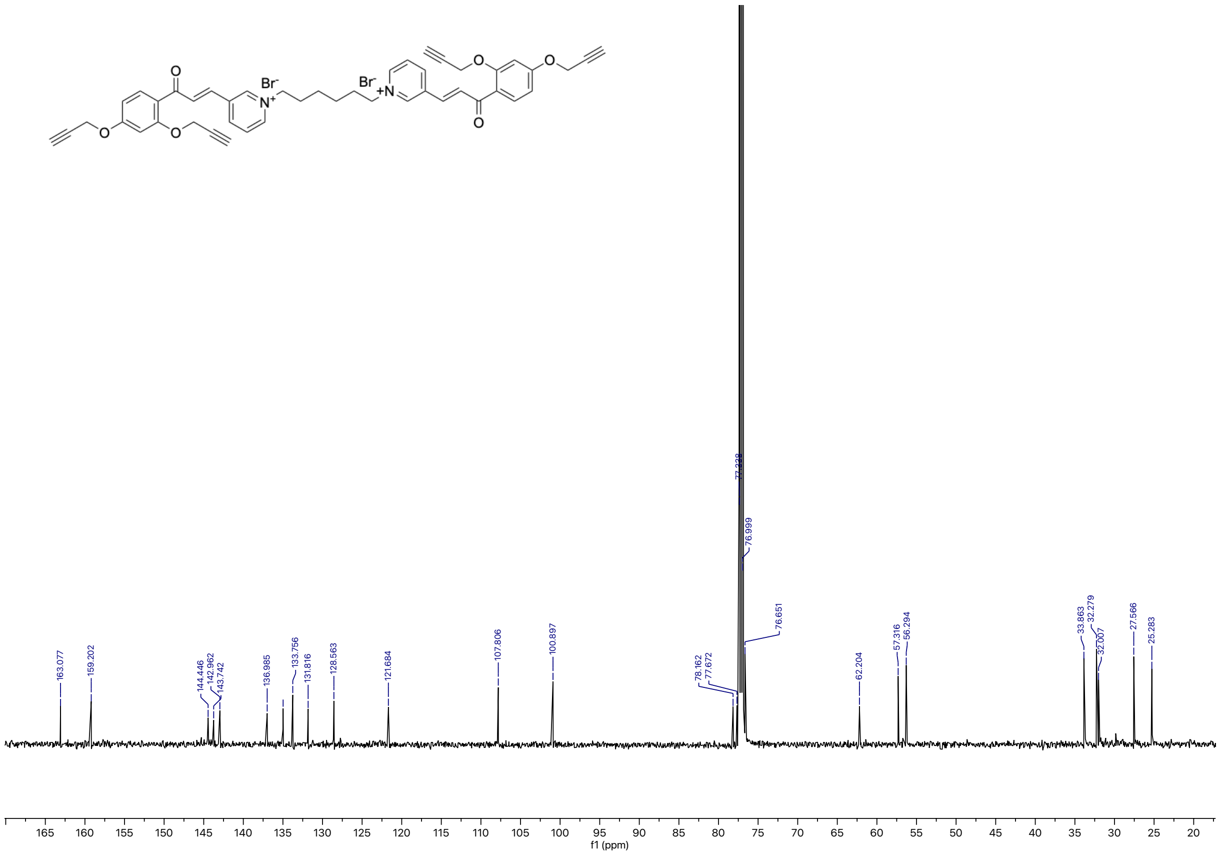


**Figure S23.** $^{13}$**C NMR spectr*um*** (151 MHz, $CDCl_3$) of compound **1**

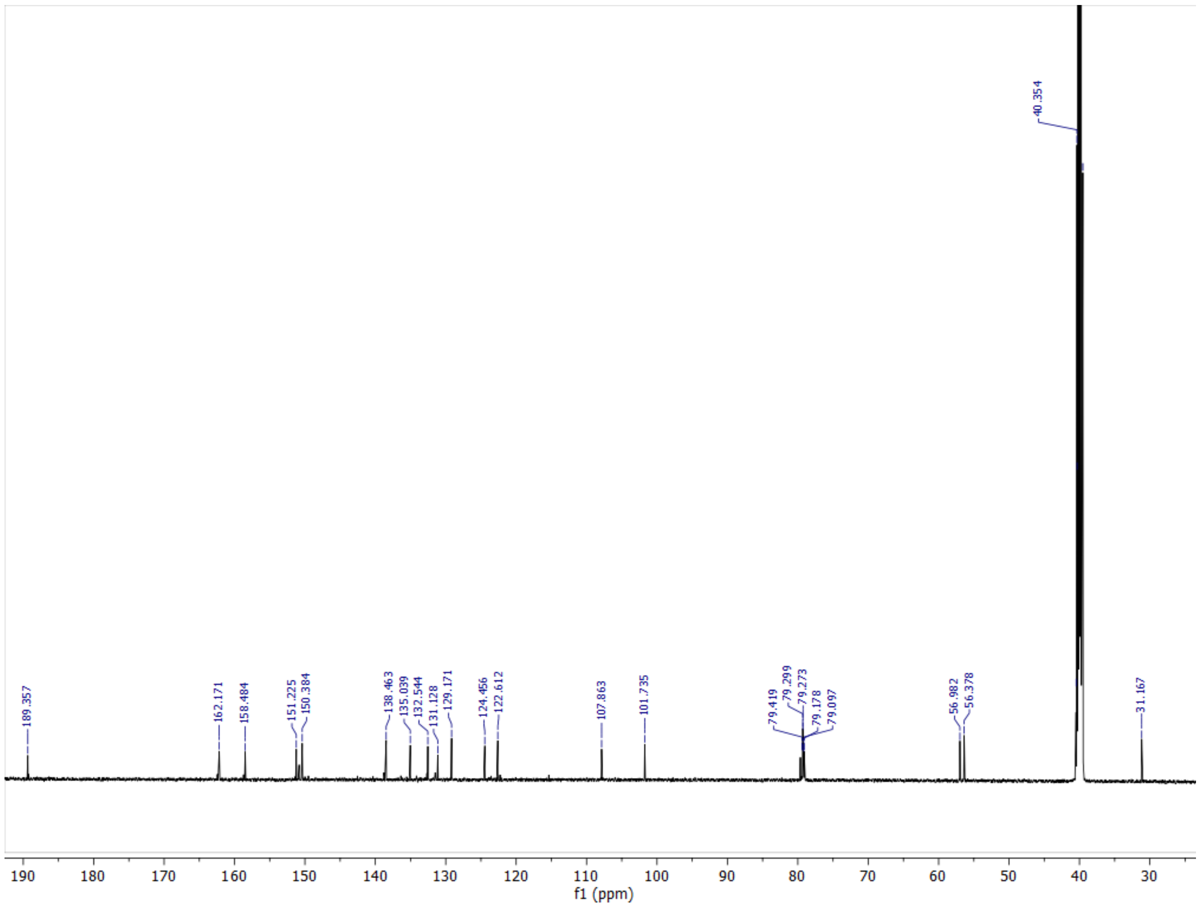


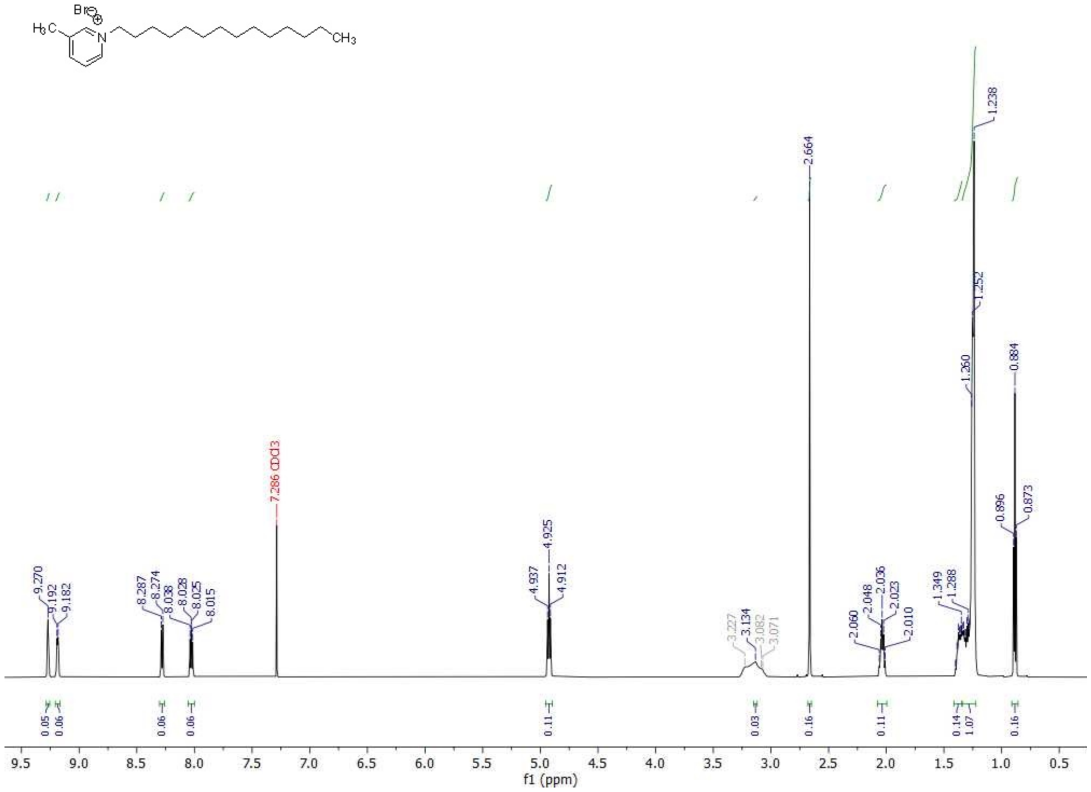


**Figure S24.** $^1$H NMR (600 MHz, $CDCl_3$) of compound **11**.

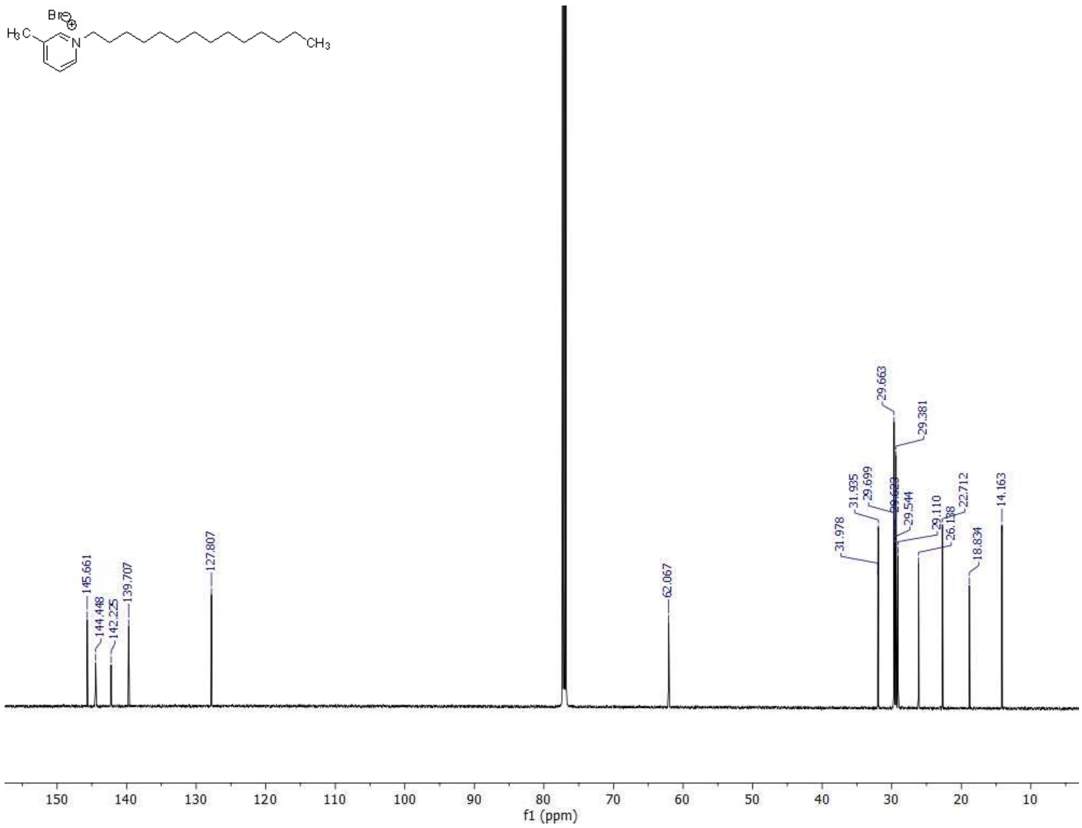


**Figure S25.** $^{13}C$ NMR (151 MHz, $CDCl_3$) of compound **11**
